# Dynamic properties of Kermack-McKendrick-like models

**DOI:** 10.1371/journal.pone.0352960

**Published:** 2026-07-14

**Authors:** Hamidou A. Diallo, Khalil Ezzinbi, Nisrine Outada, Gauthier Sallet

**Affiliations:** 1 Department of Mathematics, Faculty of Sciences Semlalia, Cadi Ayyad University, Marrakesh, Morocco; 2 Sorbonne University, 8 EDITE (ED130), IRD, UMMISCO, Bondy, Paris, France; 3 Département Institut Élie Cartan, UMR, Université de Lorraine, Metz Cedex 01, France; Lahore School of Economics, PAKISTAN

## Abstract

We rigorously analyze the dynamic properties of Kermack-McKendrick-like compartmental models for infectious diseases, extending the classical SIR framework to include exposed individuals, mild and severe infections, hospitalization, and intensive care unit (ICU) compartments. Using a 13-compartment model, we establish mathematical results on well-posedness, the basic reproduction number *R*_0_, a first integral leading to a unique final epidemic size, and the global stability of the disease-free equilibrium under permanent immunity. When temporary immunity is included, we prove the existence of an endemic equilibrium for *R*_0_ > 1. An age-stratified multi-group version of the model is also studied, demonstrating similar convergence properties and highlighting the impact of age structure on epidemic dynamics. Our results provide a rigorous mathematical framework for understanding how immunity duration, clinical progression, and age structure shape epidemic outcomes.

## 1. Introduction

Since the emergence of the COVID-19 pandemic, there has been a renewed surge of interest in compartmental models, particularly those inspired by the seminal work of Kermack and McKendrick [1]. These “Kermack-McKendrick-like” models have been pivotal in elucidating the transmission dynamics of infectious diseases, with a vast number of publications ranging from applications of the classical Susceptible-Infected-Removed (SIR) framework to sophisticated variants capturing complex epidemiological features [2–13]. The COVID-19 pandemic has exposed critical gaps in the mathematical epidemiology literature: while classical results for low-dimensional SIR and SEIR models are well established, rigorous analysis of high-dimensional systems incorporating clinical progression, hospitalization, and ICU dynamics remains sparse. In particular, the extension of fundamental results — well-posedness, basic reproduction number, final epidemic size, and endemic equilibrium — to models with Erlang-distributed sojourn times and age-stratified contact patterns has not been carried out in full generality. This paper addresses these gaps systematically. While only a subset of these references strictly adheres to the Kermack-McKendrick framework, their diversity motivates a rigorous analysis of their common dynamic properties.

We define Kermack-McKendrick-like compartmental models by the following two structural characteristics:

**H1 No demographic turnover and constant population.** There are no births, no immigration, and no natural (background, disease-independent) deaths. Disease-induced mortality, if present, is not tracked separately: deceased individuals are counted within the Removed compartment *R*, consistent with the original Kermack–McKendrick convention [1] where *R* aggregates both recovered and deceased individuals. Under H1, the conservation law
  $$\begin{array}{c} N=S+E+I+I_{s}+I_{m}+I_{h}+I_{icu}+H_{1}+H_{2}+H_{icu}+{ICU}_{1}+{ICU}_{2}+R=const \end{array}$$
  follows immediately by summing all equations of system (5) (verified in Theorem 3.1: $\dot{N}=0$). Population conservation is therefore a *consequence* of H1, not an independent postulate; we state it explicitly as part of H1 because it is used throughout the analysis. This short-timescale assumption is standard for epidemic models in which demographic turnover is negligible over the epidemic horizon [3,7].
**H2 Directed Acyclic Graph (DAG) structure** (permanent immunity, θ=0). When waning immunity is absent, the flow graph of the model has no directed cycle: clinical progression is strictly unidirectional from *S* through the infectious and clinical compartments to *R*. Under H1 and permanent immunity, no individual can return to an earlier compartment, so the DAG property holds automatically. When temporary immunity is introduced (θ>0, Section 4), the arc R→S creates a cycle and H2 is *dropped*; the infectious-to-clinical sub-graph remains acyclic, which is the only property required for the proofs of the final epidemic size (Theorem 3.3 and equation (28)).

These conditions ensure that the models focus on disease transmission over short epidemic timescales, typically excluding long-term demographic effects.

To illustrate the scope of Kermack-McKendrick-like models, we highlight several variants from the literature. The classical SIR model [1] divides the population into Susceptible (*S*), Infected (*I*), and Removed (*R*) compartments, with *R* encompassing both recovered and deceased individuals. Extensions like SIRD models separate deceased hosts into a distinct *D* compartment, maintaining dynamic equivalence to SIR by aggregating *R* and *D* [14–16]. SEIR models introduce an Exposed (*E*) compartment for latent infections [17], while SLIAR models, as proposed by F. Brauer [18], include Latent (*L*), Asymptomatic (*A*), and Symptomatic (*I*) compartments, with variations incorporating quarantine or treatment [1,19–21]. Multigroup models further account for population heterogeneity, such as age or contact patterns, with examples including age-stratified SEIR models [17,22] and complex flow graphs with Erlang-distributed sojourn times to model hospital stays [13,23]. These models consistently satisfy conditions H1–H2 (no demographic turnover, DAG structure), justifying their inclusion in our analysis.

Beyond these standard Markovian frameworks, an important stream of recent work has studied compartmental models in which sojourn times are *non-exponential*, leading to non-Markovian dynamics whose natural mathematical formulation involves Volterra-type integro-differential equations (see [24,25] and references therein). Basnarkov et al. [26] developed a non-Markovian SIR model for COVID-19 in which the infectious period follows a general distribution, showing that memory effects significantly alter the epidemic peak and timing compared to the standard exponential assumption. Granger et al. [24] introduced a four-compartment epidemic model with *retarded* (memory) transition rates expressed as convolution integrals, and derived the basic reproduction number and epidemic threshold for arbitrary sojourn distributions. The same group [25] extended this framework to stochastic compartmental models on complex networks with mortality, obtaining explicit formulas for ${ℛ}_{0}$ and endemic equilibria under Gamma-distributed sojourn times. In a complementary direction, Pastor-Satorras and Vespignani [27] established that heterogeneous contact structure in complex networks — precisely the kind of heterogeneity we model through distinct transmission rates $\beta_{I},\beta_{s},\beta_{m}$ and the age-stratified contact matrix $C=(c_{ij})$ — can qualitatively change epidemic thresholds and endemic states relative to homogeneous mixing models.

Our work is positioned at the intersection of these two streams: we exploit the *linear chain trick* [28,29] to represent Erlang-distributed sojourn times as an exact finite-dimensional ODE system (Proposition 2.1), recovering the tractability of Markovian models while retaining the non-Markovian sojourn time distribution of [24–26]. The ODE form enables explicit closed-form results for ${ℛ}_{0}$, final epidemic size, and local stability that are not available in full generality for integro-differential systems. The structured heterogeneity of our age-stratified model connects to the network epidemiology of [27] through the spectral formula ${ℛ}_{0}=\alpha (q_{I},q_{s},q_{m})\;\rho (diag(S^{0})C)$.

Several recent contributions [30–33] study related epidemic frameworks; we note, however, that these works incorporate population dynamics (births and deaths) or non-DAG transfer graphs, placing them outside the H1–H2 framework analyzed here. Our results are therefore complementary, addressing the short-timescale epidemic regime in which demographic effects are negligible.

This paper focuses on a specific class of Kermack-McKendrick-like models comprising 13 compartments: Susceptible (*S*), Exposed (*E*), Infectious (*I*), Mild Infectious ($I_{m}$), Severe Infectious ($I_{s}$), Hospitalized ($I_{h},H_{1},H_{2}$), ICU ($I_{\text{icu}},H_{\text{icu}},ICU_{1},ICU_{2}$), and Removed (*R*). Transitions are governed by compartment-specific transmission rates $\beta_{I},\beta_{s},\beta_{m}$, progression probabilities ($\pi_{1},\pi_{2}$), and Erlang-distributed sojourn times for hospital and ICU stays, as adapted from [3]. We analyze two variants: a non-age-stratified model and a multigroup age-stratified model, as used in [12,13,22]. Our objectives include deriving the basic reproduction number *R*_0_, establishing a final epidemic size relation using the first integral, analyzing the stability of the disease-free equilibrium, and proving the existence of an endemic equilibrium under temporary immunity.

Key results of this study include:

- **Convergence to Disease-Free Equilibrium (DFE)**: We prove that all trajectories in the non-age-stratified model with permanent immunity converge to a DFE, with a unique final size $S_{\infty }$ determined by a first integral.
- **Endemic Equilibrium with Temporary Immunity**: For the model with temporary immunity, we demonstrate the existence of an endemic equilibrium when *R*_0_ > 1, indicating persistent disease presence.
- **Age-Stratified Dynamics**: In the multigroup model, we show convergence to a DFE with ${ℛ}_{0}(S_{\infty })<1$, highlighting the impact of age-structured contact patterns.

These results provide important insights into how immunity assumptions fundamentally alter disease dynamics, with significant implications for public health interventions. The mathematical framework developed here offers tools for analyzing complex, high-dimensional epidemiological models while maintaining biological interpretability. Our work bridges theoretical analysis with practical model implementation, contributing to both mathematical epidemiology and public health decision-making.

The paper is organized as follows: Section 2 details the model compartments and transitions. Section 3 analyzes the non-age-stratified model, deriving *R*_0_ and the final size. Section 4 introduces temporary immunity and proves the existence of an endemic equilibrium. Section 5 examines the age-stratified model, focusing on multigroup dynamics. Section 7 summarizes findings and discusses model limitations and future directions.

## 2. Preliminaries

The model analyzed in this paper is directly adapted from the compartmental structure introduced in [3] and subsequently used in [6,9,11–13] to study the transmission dynamics of SARS-CoV-2 in France. It is therefore grounded in a well-documented epidemiological application, and its structural assumptions reflect the established clinical course of COVID-19. We justify each assumption in turn.

H1 (No demographic turnover). COVID-19 epidemic waves typically unfold over timescales of weeks to a few months, during which demographic processes have negligible effect on disease dynamics. This is consistent with the short-term modeling framework of [3,7]. Since the total population is constant by H1, we write *N* throughout instead of *N*(0), with the understanding that *N* = *N*(0) is fixed at its initial value.

H2 (DAG structure). The directed acyclic graph structure reflects the one-way clinical progression of COVID-19: susceptible individuals become exposed, then infectious, then either recover or require escalating levels of care. The only exception is temporary immunity (Section 4), where recovered individuals may return to the susceptible pool at rate θ, consistent with observed waning immunity [2].

The models, adapted from [3], comprise 13 compartments:

- *S*: Susceptible individuals.
- *E*: Exposed (infected but not yet infectious).
- *I*: Pre-symptomatic infectious individuals (infectious but not yet clinically differentiated).
- $I_{m}$: Mildly infectious individuals.
- $I_{s}$: Severely infectious individuals.
- $I_{h}$: Severely infectious individuals awaiting hospital admission (pre-admission transition stage, duration $1/\gamma_{I_{h}}$).
- $H_{1},H_{2}$: Successive general ward stages (Erlang-2 structure, mean total stay $2/\gamma_{H}\approx 11.8$ days [3]).
- *I*_icu_: Severely infectious individuals awaiting ICU admission (duration $1/\gamma_{I_{\text{icu}}}$).
- *H*_icu_: Ward-to-ICU clinical escalation stage (patients initially admitted to general ward who deteriorate and require intensive care, duration $1/\gamma_{H_{\text{icu}}}$).
- ${ICU}_{1},{ICU}_{2}$: Successive ICU stay stages (Erlang-2 structure, mean total stay $2/\gamma_{\text{icu}}\approx 15.0$ days [3]).
- *R*: Removed (recovered or deceased).

The 13-compartment structure serves a dual purpose. The upstream sub-system — comprising *S*, *E*, *I*, $I_{m}$, $I_{s}$ — governs all transmission dynamics and is solely responsible for *R*_0_, the final epidemic size, and the endemic equilibrium condition. In this sense, the theoretical results of Sections 3 and 4 are structurally equivalent to those of a reduced SEIR-type model: the hospitalization and ICU compartments are downstream of the transmission chain (by H2) and do not feed back into it. Their inclusion is therefore not required for the core mathematical results, but is essential for projecting hospital and ICU occupancy — the primary clinical indicators driving public health decisions during COVID-19 [3].

Transitions are governed by:

- Transmission from *S* to *E* at force of infection $\beta_{I}I+\beta_{s}I_{s}+\beta_{m}I_{m}$, where $\beta_{I},\beta_{s},\beta_{m}>0$ are the compartment-specific transmission rates of pre-symptomatic (*I*), severely symptomatic ($I_{s}$), and mildly symptomatic/asymptomatic ($I_{m}$) individuals respectively.
- Progression from *E* to *I* at rate $\gamma_{E}>0$.
- From *I* to $I_{s}$ (probability $\pi_{1}\in [0,1]$) or $I_{m}$ (probability $1-\pi_{1}$) at rate $\gamma_{I}>0$.
- From $I_{s}$ to hospital ($I_{h}$, probability $\pi_{2}\in [0,1]$) or ICU (*I*_icu_, probability $1-\pi_{2}$) at rate $\gamma_{1}>0$.
- Hospital stays ($H_{1},H_{2}$) follow an Erlang distribution with shape 2 and mean $\frac{2}{\gamma_{H}}$, $\gamma_{H}>0$.
- ICU stays (${ICU}_{1},{ICU}_{2}$) follow an Erlang distribution with shape 2 and mean $\frac{2}{\gamma_{\text{icu}}}$, $\gamma_{\text{icu}}>0$.

**Compartment-specific transmission rates** $\beta_{I},\beta_{s},\beta_{m}$. The three pre-hospitalization infectious compartments correspond to biologically distinct transmission profiles. Pre-symptomatic individuals (*I*) are infectious but not yet coughing significantly; He et al. [34] estimated that around 44% of SARS-CoV-2 transmissions occurred before symptom onset, with peak infectivity near day 0–1 of symptoms. Mildly symptomatic individuals ($I_{m}$) include asymptomatic and pauci-symptomatic cases; their viral loads at the time of symptom differentiation are broadly comparable to those of pre-symptomatic individuals [35]. Severely symptomatic individuals ($I_{s}$) can have higher peak viral shedding, but their mean community sojourn is short ($1/\gamma_{1}$ days) before hospital admission, and behavioral self-isolation (staying home due to illness) tends to reduce their effective contact rate [34]. We therefore formulate the model with three distinct parameters $\beta_{I},\beta_{s},\beta_{m}>0$. The well-posedness, next-generation matrix, final-size relation, and disease-free stability results are established for the full three-rate formulation. The detailed local stability analysis of the endemic equilibrium is carried out under the calibration constraint $\beta_{I}=\beta_{s}=\beta_{m}=:\beta$, which is the parameterization used in the numerical simulations.

In the numerical simulations (Section 6), we impose the calibration constraint $\beta_{I}=\beta_{s}=\beta_{m}=:\beta$. This is motivated by *identifiability*: the three rates enter ${ℛ}_{0}=S_{0}\left(\beta_{I}/\gamma_{I}+(\beta_{s}\pi_{1}+\beta_{m}(1-\pi_{1}))/\gamma_{1}\right)$ as a single compound scalar, so only this linear combination is estimable from aggregate hospitalization time-series [35]. From a network-epidemiology perspective, the three-rate formulation $\left(\beta_{I},\beta_{s},\beta_{m}\right)$ is analogous to the heterogeneous transmission rates arising from degree-distributed contact networks studied by Pastor-Satorras and Vespignani [27]: in both settings, the effective reproduction number depends on a spectral quantity that aggregates individual transmission contributions. Separate estimation of $\beta_{I}$, $\beta_{s}$, $\beta_{m}$ requires high-resolution contact-tracing or household-study data, which lies beyond the scope of the present mathematical analysis and is listed as a future direction.

All infectious individuals differentiate into mild or severe. Compartment *I* represents the pre-symptomatic infectious stage (mean duration $1/\gamma_{I}\approx 1$ day [3]) before any clinical differentiation. At the end of this stage the epidemic trajectory *bifurcates in parallel*: with probability $\pi_{1}$ the individual becomes severely symptomatic ($I_{s}$) and with probability $1-\pi_{1}$ mildly symptomatic or asymptomatic ($I_{m}$). These are two *concurrent* clinical pathways, not a sequential progression from mild to severe: $I_{m}$ and $I_{s}$ are never connected by a transition, reflecting the clinical observation that disease severity is largely determined at symptom onset rather than developing progressively [3,7]. Mild infectious individuals ($I_{m}$) correspond to asymptomatic or pauci-symptomatic cases that largely escape clinical detection; the limiting case $\pi_{1}=0$ yields a purely asymptomatic model.

Necessity of the exposed compartment *E*. Although all exposed individuals eventually become infectious, the explicit inclusion of *E* is both biologically and mathematically necessary. Biologically, the latent period (≈4 days [3]) and the pre-symptomatic infectious period (≈1 day [3]) correspond to fundamentally different epidemiological states: exposed individuals do not transmit, while infectious individuals do. Collapsing *E* into *I* would overestimate *R*_0_. Mathematically, the initial condition *E*_0_ appears explicitly in the final size equation (28): removing *E* would alter the final size prediction whenever *E*_0_ > 0.

Exposed compartment vs. Erlang hospitalization. A natural question arises: the model uses a single Exposed compartment *E* (producing an exponential latency with CV  =  1) rather than an Erlang chain, while hospital and ICU stays use two-stage Erlang chains. This is not inconsistent, but reflects different data constraints. The mean incubation period of COVID-19 is approximately 3–5 days [2,3] with an observed CV close to 1 [36], so the exponential approximation is acceptable for the latent stage. In contrast, hospital stays of mean 11.8 days with CV  < 1 are better described by a sharper distribution; a single exponential stage would discharge ≈22% of patients within 3 days, which is clinically unrealistic. Adding a second stage reduces this to ≈8%, bringing the model in line with observed length-of-stay distributions [3,37]. An Erlang-2 Exposed chain could be added without loss of mathematical tractability, but would not change any of the theoretical results (the NGM and final size are independent of the number of Erlang stages in the upstream chain by the DAG property H2) and is not supported by a clear statistical improvement for the short incubation period.

The model describes a short-term epidemic process in a closed population with homogeneous mixing in the non-age-stratified setting. Susceptible individuals become exposed after effective contact with pre-symptomatic, mildly symptomatic/asymptomatic, or severely symptomatic infectious individuals. They then progress through a pre-symptomatic infectious stage before branching into mild/asymptomatic or severe clinical pathways. Severe cases may subsequently require general-ward or ICU care, represented by Erlang-2 clinical chains, while mild cases move directly to the removed class. Tables 1 and 2 summarize the compartments and parameters, respectively.

**Table 1:** Summary of model compartments and their clinical interpretation.

Symbol	Clinical meaning	Duration
*S*	Susceptible	—
*E*	Exposed (latent, non-infectious)	$1/\gamma_{E}\approx 4$ days
*I*	Pre-symptomatic infectious	$1/\gamma_{I}\approx 1$ day
$I_{s}$	Severely infectious (community)	$1/\gamma_{1}$ days
$I_{m}$	Mildly infectious (community)	$1/\gamma_{1}$ days
$I_{h}$	Severe case awaiting ward admission	$1/\gamma_{I_{h}}$ days
$H_{1},H_{2}$	General ward stay (Erlang-2)	mean $2/\gamma_{H}\approx 11.8$ days
*I*_icu_	Severe case awaiting ICU admission	$1/\gamma_{I_{\text{icu}}}$ days
*H*_icu_	Ward-to-ICU escalation	$1/\gamma_{H_{\text{icu}}}$ days
${ICU}_{1},{ICU}_{2}$	ICU stay (Erlang-2)	mean $2/\gamma_{\text{icu}}\approx 15.0$ days
*R*	Removed (recovered or deceased)	—

**Table 2:** Summary of model parameters with biological interpretation.

Symbol	Type	Description	Unit
$\beta_{I}$	Transmission	Pre-symptomatic transmission rate	person^−1^ day^−1^
$\beta_{s}$	Transmission	Severe symptomatic transmission rate	person^−1^ day^−1^
$\beta_{m}$	Transmission	Mild/asymptomatic transmission rate	person^−1^ day^−1^
$\gamma_{E}$	Progression	Rate E→I	day^−1^
$\gamma_{I}$	Progression	Rate $I\to I_{s}$ or $I_{m}$	day^−1^
$\gamma_{1}$	Progression	Rate $I_{s},I_{m}\to$ downstream	day^−1^
$\gamma_{I_{h}}$	Hospital	Rate $I_{h}\to H_{1}$	day^−1^
$\gamma_{H}$	Hospital	Erlang rate, general ward	day^−1^
$\gamma_{I_{\text{icu}}}$	ICU	Rate $I_{\text{icu}}\to H_{\text{icu}}$	day^−1^
$\gamma_{H_{\text{icu}}}$	ICU	Rate $H_{\text{icu}}\to {ICU}_{1}$	day^−1^
$\gamma_{\text{icu}}$	ICU	Erlang rate, ICU stages	day^−1^
$\pi_{1}$	Branching	Probability $I\to I_{s}$	dimensionless
$\pi_{2}$	Branching	Probability $I_{s}\to I_{h}$	dimensionless
θ	Immunity	Rate of immunity loss (R→S)	day^−1^
*R*_0_	Derived	Basic reproduction number	dimensionless

**Fig 1:**
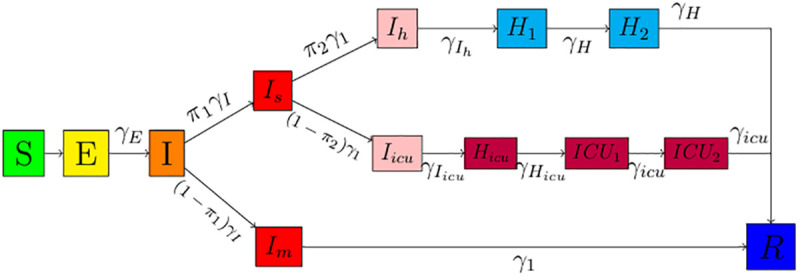
Flowchart of the model. Diagram of transitions between compartments: Susceptible (*S*) to Exposed (*E*) at force of infection $\beta_{I}I+\beta_{s}I_{s}+\beta_{m}I_{m}$, with subsequent progression through infectious, hospitalized, ICU, and Removed (*R*) compartments, governed by rates and probabilities as described [3,4].

The corresponding flowchart is shown in Fig 1.

### 2.1. The linear chain trick: Erlang Sojourn Times as an Exact ODE reformulation

**Why Erlang-2 for hospital and ICU stays.** The choice of Erlang-2 distributions is motivated by three independent arguments.

**Why two ward compartments (**$H_{1}\to H_{2}$, **Erlang-2) and not one or three?**. The model uses two successive general ward compartments $H_{1}\to H_{2}$ (and similarly ${ICU}_{1}\to {ICU}_{2}$ for intensive care). We clarify at the outset that *H*_1_, *H*_2_ (and *H*_3_ below) denote *compartments of the model*, not the modeling hypotheses of Section 2 (which carry the sans-serif labels H1, H2). The question is: why use an Erlang-2 chain (two stages) rather than a single exponential stage (Erlang-1) or three stages (Erlang-3)? The answer is quantitative:

1. *Statistical inadequacy of the exponential.* A single exponential stage produces a memoryless sojourn time with coefficient of variation CV  =  1. For a mean hospital stay of 11.8 days, this implies ≈22% of patients discharged within 3 days — clinically unrealistic. The table below compares the three leading ODE-compatible choices:

**Table:** 

Distribution	CV	% discharged <3 d (mean 11.8 d)	Assessment
Erlang-1 (exponential)	1.00	≈22%	Clinically unrealistic
Erlang-2	$1/\sqrt{2}\approx 0.71$	≈8%	Consistent with data [3,37]
Erlang-3	$1/\sqrt{3}\approx 0.58$	≈3%	Adds one ODE per chain; unjustified by data

Erlang-2 is therefore the *minimal* ODE-compatible extension beyond exponential that substantially reduces variance, at the cost of only one additional equation per clinical sub-chain.

2. *Empirical fit.* COVID-19 hospital and ICU length-of-stay distributions are unimodal with CV  < 1, inconsistent with the exponential but consistent with Erlang-2 [3,37]. Higher-order Erlang chains (k≥3) are not supported by available length-of-stay data [3,37].
3. *ODE tractability and exact equivalence with the convolution framework.* Among distributions with CV  < 1, Erlang distributions provide a standard and parsimonious class that admits an exact finite-dimensional ODE reformulation via the linear chain trick (Proposition 2.1). More general distributions (Weibull, log-normal) would require integro-differential equations with memory kernels, as studied by Granger et al. in the retarded-transition-rate framework of [24,25]. Those works obtain ${ℛ}_{0}$ and endemic equilibria for *arbitrary* sojourn distributions, at the cost of working in an infinite-dimensional function space. Our Erlang-2 chain is the exact ODE realization of the special case where the memory kernel is a Gamma-$\left(2,\gamma_{H}\right)$ density (see Proposition 2.1 and its proof by variation of constants): the two frameworks are mathematically equivalent for this kernel, but our ODE formulation yields the explicit closed-form results of Sections 3–5 that are not available in full generality from the integro-differential approach. Erlang-2 achieves the best statistical improvement over the exponential at minimal model complexity (one additional ODE per clinical chain).

**The linear chain trick: exact equivalence with integro-differential equations**. Erlang-distributed sojourn times are, in principle, *non-Markovian*: they correspond to residence-time distributions with memory, whose natural formulation is a Volterra-type integro-differential equation [24, 25]. The key insight, established in [28,29], is that this non-Markovian system admits an *exact* finite-dimensional ODE reformulation via the *linear chain trick*.

**Proposition 2.1 (Linear chain trick**, [28,29]). *Let Z(t) represent the total occupancy of a compartment with Erlang-2 sojourn time distribution, mean* 2/γ*, and exogenous inflow u(t). The following two formulations are mathematically equivalent:*

- ***Integro-differential form:***
  $$\begin{array}{c} \dot{Z}(t)=u(t)-\int_{0}^{t}\gamma^{2}s\;e^{-\gamma s}\;u(t-s)\;ds, \end{array}$$ (1)
  *where* $k_{2}(s)=\gamma^{2}s\;e^{-\gamma s}$ *is the Erlang-2 probability density function (residence-time kernel).*
- ***ODE chain form:***
  $$\begin{array}{c} {\dot{X}}_{1}=u(t)-\gamma X_{1},\;{\dot{X}}_{2}=\gamma X_{1}-\gamma X_{2},\;Z=X_{1}+X_{2}. \end{array}$$ (2)
  *Both formulations produce identical output Z(t) for any input u(t) and zero initial conditions. The sojourn time of an individual entering the chain (2) is distributed as* Erlang(2,γ)*, with mean* 2/γ *and variance* $2/\gamma^{2}$.

**Proof 2.2** *We use the method of variation of constants (variation of parameters) to solve the ODE chain explicitly, then read off the equivalence with the integro-differential form and the sojourn time distribution.*

***Step 1: Solution of the first stage by variation of constants.*** *The equation* ${\dot{X}}_{1}=u(t)-\gamma X_{1}$ *is a first-order linear ODE. The associated homogeneous equation* ${\dot{X}}_{1}=-\gamma X_{1}$ *has the fundamental solution* $\varphi (t)=e^{-\gamma t}$*. By variation of constants, we set* $X_{1}(t)=c(t)\;e^{-\gamma t}$ *and require:*


$$\begin{array}{c} \dot{c}(t)\;e^{-\gamma t}=u(t)\;⟹\;c(t)=c(0)+\int_{0}^{t}e^{\gamma \tau }\;u(\tau )\;d\tau . \end{array}$$

*With zero initial condition X*_1_(0) = 0 (so *c*(0)=0):


$$\begin{array}{c} X_{1}(t)=\int_{0}^{t}e^{-\gamma (t-\tau )}\;u(\tau )\;d\tau . \end{array}$$ (3)

***Step 2: Solution of the second stage by variation of constants.*** *The equation* ${\dot{X}}_{2}=\gamma X_{1}(t)-\gamma X_{2}$ *is driven b*y *X*_1_(*t*) *computed in (3). Its homogeneous fundamental solution is again* $e^{-\gamma t}$*. Setting* $X_{2}(t)=d(t)\;e^{-\gamma t}$ *and requiring* $\dot{d}(t)\;e^{-\gamma t}=\gamma X_{1}(t)$*:*


$$\begin{array}{c} d(t)=d(0)+\gamma \int_{0}^{t}e^{\gamma s}\;X_{1}(s)\;ds. \end{array}$$

*With X*_2_(0) = 0, *substituting (3) into the above:*


$$\begin{array}{c} X_{2}(t)=\gamma e^{-\gamma t}\int_{0}^{t}e^{\gamma s}\left[\int_{0}^{s}e^{-\gamma (s-\tau )}\;u(\tau )\;d\tau \right]ds. \end{array}$$

*Exchanging the order of integration (Fubini;* 0≤τ≤s≤t*):*


$$\begin{array}{c} X_{2}(t)=\gamma e^{-\gamma t}\int_{0}^{t}u(\tau )\underset{=\;e^{\gamma \tau }(t-\tau )}{\underbrace{\int_{\tau }^{t}e^{\gamma s}\cdot e^{-\gamma (s-\tau )}\;ds}}\;d\tau =\gamma \int_{0}^{t}(t-\tau )\;e^{-\gamma (t-\tau )}\;u(\tau )\;d\tau . \end{array}$$

***Step 3: Total occupancy and the occupancy kernel.*** *Adding both stages:*


$$\begin{array}{c} Z(t)=X_{1}(t)+X_{2}(t)=\int_{0}^{t}\underset{=:\;h(t-\tau )}{\underbrace{[1+\gamma (t-\tau )]e^{-\gamma (t-\tau )}}}\;u(\tau )\;d\tau . \end{array}$$ (4)

*The kernel* $h(\sigma )=(1+\gamma \sigma )e^{-\gamma \sigma }$ *is the survival function of the* Erlang(2,γ) *distribution*:


$$\begin{array}{c} h(\sigma )=P(T>\sigma )=1-F_{Erl(2,\gamma )}(\sigma ), \end{array}$$

*where* $F_{Erl(2,\gamma )}(\sigma )=1-(1+\gamma \sigma )e^{-\gamma \sigma }$ *is the standard Erlang-2 CDF. Hence* equation (4) *is the occupancy formula* $Z(t)=\int_{0}^{t}P(T>t-\tau )\;u(\tau )\;d\tau$*: the number of individuals present at time t equals the sum over all past arrivals* u(τ)dτ *of the probability* h(t−τ) *of still being in the chain at time t.*

***Step 4: Equivalence with the integro-differential form.*** *Differentiating (4) with respect to t and using h*(0) = 1, $\dot{h}(\sigma )=-\gamma^{2}\sigma \;e^{-\gamma \sigma }=-k_{2}(\sigma )$:


$$\begin{array}{c} \dot{Z}(t)=h(0)\;u(t)+\int_{0}^{t}\dot{h}(t-\tau )\;u(\tau )\;d\tau =u(t)-\int_{0}^{t}\underset{k_{2}(t-\tau )}{\underbrace{\gamma^{2}(t-\tau )e^{-\gamma (t-\tau )}}}\;u(\tau )\;d\tau , \end{array}$$

*which is exactly the integro-differential form (1). The equivalence is therefore a direct consequence of the variation-of-constants solution and the identity* $-\dot{h}=k_{2}=f_{Erl(2,\gamma )}$.

***Step 5: Sojourn time distribution.*** *Set u*(*t*)=0 *and consider a single individual entering X*_1_ at *t* = 0 (i.e., *X*_1_(0)=1, *X*_2_(0)=0). *Its sojourn in stage* 1 *is* $T_{1}\sim Exp(\gamma )$ (*constant departure rate* γ*); similarly, its sojourn in stage 2 is* $T_{2}\sim Exp(\gamma )$*, independent of T*_1_*. The total time* $T=T_{1}+T_{2}$ *has density:*


$$\begin{array}{c} f_{T}(\sigma )=(\gamma e^{-\gamma \sigma })*(\gamma e^{-\gamma \sigma })=\gamma^{2}\sigma \;e^{-\gamma \sigma }=k_{2}(\sigma ),\;\sigma \geq 0, \end{array}$$

*confirming* T~Erlang(2,γ)*. The survival function is* $P(T>\sigma )=(1+\gamma \sigma )e^{-\gamma \sigma }=h(\sigma )$*, consistent with Step 3. The moments follow:*


$$\begin{array}{c} 𝔼[T]=\frac{2}{\gamma },\;Var(T)=\frac{2}{\gamma^{2}},\;CV(T)=\frac{1}{\sqrt{2}}\approx 0.71. \end{array}$$

**Application to the two clinical chains of model (5).**

**General ward chain** $\left(I_{h}\to H_{1}\to H_{2}\right)$. The inflow to the ward sub-system is $u_{H}(t)=\gamma_{I_{h}}I_{h}(t)$, corresponding to severely infectious individuals admitted to the general ward. By (3)–(4) applied with $\gamma =\gamma_{H}$:


$$\begin{array}{cc} H_{1}(t) & =e^{-\gamma_{H}t}H_{1}(0)+\int_{0}^{t}e^{-\gamma_{H}(t-s)}\;\gamma_{I_{h}}I_{h}(s)\;ds,\\ H_{2}(t) & =e^{-\gamma_{H}t}H_{2}(0)+\gamma_{H}e^{-\gamma_{H}t}\int_{0}^{t}e^{\gamma_{H}s}H_{1}(s)\;ds\\ & =e^{-\gamma_{H}t}H_{2}(0)+\gamma_{H}\int_{0}^{t}(t-s)\;e^{-\gamma_{H}(t-s)}\;\gamma_{I_{h}}I_{h}(s)\;ds\;+\;\text{(transient from }H_{1}(0)\text{)}. \end{array}$$

The total ward occupancy $W(t)=H_{1}(t)+H_{2}(t)$ satisfies, for zero initial conditions:


$$\begin{array}{c} W(t)=\int_{0}^{t}[1+\gamma_{H}(t-s)]e^{-\gamma_{H}(t-s)}\;\gamma_{I_{h}}I_{h}(s)\;ds, \end{array}$$

and its dynamics obey the integro-differential equation:


$$\begin{array}{c} \dot{W}(t)=\gamma_{I_{h}}I_{h}(t)-\int_{0}^{t}\gamma_{H}^{2}(t-s)\;e^{-\gamma_{H}(t-s)}\;\gamma_{I_{h}}I_{h}(s)\;ds. \end{array}$$

The sojourn time in the ward is $Erlang(2,\gamma_{H})$ with mean $2/\gamma_{H}\approx 11.8$ days and $CV=1/\sqrt{2}\approx 0.71$ (consistent with French COVID-19 hospitalization data [3,37]).

ICU chain $I_{icu}\to H_{icu}\to {ICU}_{1}\to {ICU}_{2}$. The sub-chain $\left({ICU}_{1},{ICU}_{2}\right)$ receives inflow $u_{ICU}(t)=\gamma_{H_{icu}}H_{icu}(t)$ from patients who deteriorate from the general ward to intensive care. By (3)–(4) with $\gamma =\gamma_{icu}$:


$$\begin{array}{c} {ICU}_{1}(t)=e^{-\gamma_{icu}t}{ICU}_{1}(0)+\int_{0}^{t}e^{-\gamma_{icu}(t-s)}\;\gamma_{H_{icu}}H_{icu}(s)\;ds,\\ {ICU}_{2}(t)=e^{-\gamma_{icu}t}{ICU}_{2}(0)+\gamma_{icu}\int_{0}^{t}(t-s)\;e^{-\gamma_{icu}(t-s)}\;\gamma_{H_{icu}}H_{icu}(s)\;ds\;+\;\text{(transient)}, \end{array}$$

and the total ICU occupancy $U(t)={ICU}_{1}(t)+{ICU}_{2}(t)$ satisfies:


$$\begin{array}{c} \dot{U}(t)=\gamma_{H_{icu}}H_{icu}(t)-\int_{0}^{t}\gamma_{icu}^{2}(t-s)\;e^{-\gamma_{icu}(t-s)}\;\gamma_{H_{icu}}H_{icu}(s)\;ds. \end{array}$$

The ICU sojourn time is $Erlang(2,\gamma_{icu})$ with mean $2/\gamma_{icu}\approx 15.0$ days and $CV=1/\sqrt{2}$.

**Conclusion.** In both chains, the ODE system of model (5) is the *exact* variation-of-constants representation of the corresponding Volterra integro-differential equation with Erlang-2 kernel. The transition ${ICU}_{1}\to {ICU}_{2}$ (and $H_{1}\to H_{2}$) is therefore not a clinical event but a mathematical stage: both stages together constitute a single continuous episode whose total duration is Erlang-2 distributed.

## 3 Model without age stratification

We first analyze the baseline version of the model without demographic or age structure. This case illustrates the essential mathematical features of the framework, including well-posedness, the basic reproduction number, and the final epidemic size.

The resulting system from the figure shown in Fig 1 is:


$$\begin{array}{c} \left\{ \begin{array}{c} \dot{S}=-(\beta_{I}I+\beta_{s}I_{s}+\beta_{m}I_{m})S,\\ \dot{E}=(\beta_{I}I+\beta_{s}I_{s}+\beta_{m}I_{m})S-\gamma_{E}E,\\ \dot{I}=\gamma_{E}E-\gamma_{I}I,\\ {\dot{I}}_{s}=\pi_{1}\gamma_{I}I-\gamma_{1}I_{s},\\ {\dot{I}}_{m}=(1-\pi_{1})\gamma_{I}I-\gamma_{1}I_{m},\\ {\dot{I}}_{h}=\pi_{2}\gamma_{1}I_{s}-\gamma_{I_{h}}I_{h},\\ {\dot{I}}_{\text{icu}}=(1-\pi_{2})\gamma_{1}I_{s}-\gamma_{I_{\text{icu}}}I_{\text{icu}},\\ {\dot{H}}_{1}=\gamma_{I_{h}}I_{h}-\gamma_{H}H_{1},\\ {\dot{H}}_{2}=\gamma_{H}H_{1}-\gamma_{H}H_{2},\\ {\dot{H}}_{\text{icu}}=\gamma_{I_{\text{icu}}}I_{\text{icu}}-\gamma_{H_{\text{icu}}}H_{\text{icu}},\\ {\dot{ICU}}_{1}=\gamma_{H_{\text{icu}}}H_{\text{icu}}-\gamma_{\text{icu}}ICU_{1},\\ {\dot{ICU}}_{2}=\gamma_{\text{icu}}ICU_{1}-\gamma_{\text{icu}}ICU_{2},\\ \dot{R}=\gamma_{1}I_{m}+\gamma_{H}H_{2}+\gamma_{\text{icu}}ICU_{2}, \end{array}\right. \end{array}$$ (5)

### 3.1 Well-posedness of the model

To ensure the mathematical validity of the non-age-stratified Kermack-McKendrick-like model, we analyze its well-posedness, establishing the existence, uniqueness, non-negativity, and boundedness of solutions. The model is described by the following system of ordinary differential equations:


$$\begin{array}{c} \dot{S}=-(\beta_{I}I+\beta_{s}I_{s}+\beta_{m}I_{m})S, \end{array}$$ (6)

$$\begin{array}{c} \dot{E}=(\beta_{I}I+\beta_{s}I_{s}+\beta_{m}I_{m})S-\gamma_{E}E, \end{array}$$ (7)

$$\begin{array}{c} \dot{I}=\gamma_{E}E-\gamma_{I}I, \end{array}$$ (8)

$$\begin{array}{c} {\dot{I}}_{s}=\pi_{1}\gamma_{I}I-\gamma_{1}I_{s}, \end{array}$$ (9)

$$\begin{array}{c} {\dot{I}}_{m}=(1-\pi_{1})\gamma_{I}I-\gamma_{1}I_{m}, \end{array}$$ (10)

$$\begin{array}{c} {\dot{I}}_{h}=\pi_{2}\gamma_{1}I_{s}-\gamma_{I_{h}}I_{h}, \end{array}$$ (11)

$$\begin{array}{c} {\dot{I}}_{\text{icu}}=(1-\pi_{2})\gamma_{1}I_{s}-\gamma_{I_{\text{icu}}}I_{\text{icu}}, \end{array}$$ (12)

$$\begin{array}{c} {\dot{H}}_{1}=\gamma_{I_{h}}I_{h}-\gamma_{H}H_{1}, \end{array}$$ (13)

$$\begin{array}{c} {\dot{H}}_{2}=\gamma_{H}H_{1}-\gamma_{H}H_{2}, \end{array}$$ (14)

$$\begin{array}{c} {\dot{H}}_{\text{icu}}=\gamma_{I_{\text{icu}}}I_{\text{icu}}-\gamma_{H_{\text{icu}}}H_{\text{icu}}, \end{array}$$ (15)

$$\begin{array}{c} {\dot{ICU}}_{1}=\gamma_{H_{\text{icu}}}H_{\text{icu}}-\gamma_{\text{icu}}ICU_{1}, \end{array}$$ (16)

$$\begin{array}{c} {\dot{ICU}}_{2}=\gamma_{\text{icu}}ICU_{1}-\gamma_{\text{icu}}ICU_{2}, \end{array}$$ (17)

$$\begin{array}{c} \dot{R}=\gamma_{1}I_{m}+\gamma_{H}H_{2}+\gamma_{\text{icu}}ICU_{2}, \end{array}$$ (18)

where *S*, *E*, *I*, $I_{s}$, $I_{m}$, $I_{h}$, *I*_icu_, *H*_1_, *H*_2_, *H*_icu_, *ICU*_1_, *ICU*_2_, and *R* represent the susceptible, exposed, infectious, severe infectious, mild infectious, hospitalized, ICU-admitted, and removed compartments, respectively. Parameters $\beta_{I},\beta_{s},\beta_{m}>0$, $\gamma_{E},\gamma_{I},\gamma_{1},\gamma_{I_{h}},\gamma_{H},\gamma_{I_{\text{icu}}},\gamma_{H_{\text{icu}}},\gamma_{\text{icu}}>0$, and $0<\pi_{1},\pi_{2}<1$ govern transmission, progression, and branching probabilities.

Let the state vector be $𝐱=(S,E,I,I_{s},I_{m},I_{h},I_{\text{icu}},H_{1},H_{2},H_{\text{icu}},ICU_{1},ICU_{2},R)\in {\mathbb{R}}^{13}$. The biologically meaningful domain is:


$$\begin{array}{c} \Omega =\left\{𝐱\in {\mathbb{R}}_{\geq 0}^{13}|S+E+I+I_{s}+I_{m}+I_{h}+I_{\text{icu}}+H_{1}+H_{2}+H_{\text{icu}}+ICU_{1}+ICU_{2}+R=N\right\}, \end{array}$$

where *N* > 0 is the constant total population (H1), and initial conditions 𝐱(0)∈Ω.

**Theorem 3.1.** *For any initial condition* 𝐱(0)∈Ω*, the system (6)–(18) admits a unique solution* 𝐱(t)∈Ω *defined for all* t≥0*. Moreover, the solution is non-negative and bounded, with* 0≤S(t),E(t),…,R(t)≤N*.*

**Proof 3.2***. The system can be written as* $\dot{𝐱}=𝐟(𝐱)$*, where* $𝐟:{\mathbb{R}}^{13}\to {\mathbb{R}}^{13}$ *is defined by the right-hand sides of* (6)–(18). *The function* 𝐟 *consists of linear and bilinear terms (e.g.,* $-\beta_{I}SI$*,* $-\beta_{s}SI_{s}$*,* $-\beta_{m}SI_{m}$*). Since* 𝐟 *is continuously differentiable on* ${\mathbb{R}}^{13}$*, it is locally Lipschitz continuous in any bounded region, including* Ω*. By the Cauchy-Lipschitz theorem, there exists a unique local solution for any* 𝐱(0)∈Ω*. To ensure global existence, we demonstrate boundedness below.*

*To show non-negativity, consider the behavior of each compartment on the boundary of* ${\mathbb{R}}_{\geq 0}^{13}$*. If S*(*t*) = 0, *then* $\dot{S}=0$, *so S*(*t*) *remains non-negative. If E*(*t*) = 0, *then* $\dot{E}=(\beta_{I}I+\beta_{s}I_{s}+\beta_{m}I_{m})S\geq 0$, *since* $S,I,I_{s},I_{m}\geq 0$. *Similarly*:

- *I*(*t*) = 0: $\dot{I}=\gamma_{E}E\geq 0$,
- $I_{s}(t)=0$: ${\dot{I}}_{s}=\pi_{1}\gamma_{I}I\geq 0$,
- $I_{m}(t)=0$: ${\dot{I}}_{m}=(1-\pi_{1})\gamma_{I}I\geq 0$,
- $I_{h}(t)=0$: ${\dot{I}}_{h}=\pi_{2}\gamma_{1}I_{s}\geq 0$,
- *I*_icu_(*t*) = 0: ${\dot{I}}_{\text{icu}}=(1-\pi_{2})\gamma_{1}I_{s}\geq 0$,
- *H*_1_(*t*) = 0: ${\dot{H}}_{1}=\gamma_{I_{h}}I_{h}\geq 0$,
- *H*_2_(*t*) = 0: ${\dot{H}}_{2}=\gamma_{H}H_{1}\geq 0$,
- *H*_icu_(*t*) = 0: ${\dot{H}}_{\text{icu}}=\gamma_{I_{\text{icu}}}I_{\text{icu}}\geq 0$,
- *ICU*_1_(*t*) = 0: ${\dot{ICU}}_{1}=\gamma_{H_{\text{icu}}}H_{\text{icu}}\geq 0$,
- *ICU*_2_(*t*) = 0: ${\dot{ICU}}_{2}=\gamma_{\text{icu}}ICU_{1}\geq 0$,
- *R*(*t*) = 0: $\dot{R}=\gamma_{1}I_{m}+\gamma_{H}H_{2}+\gamma_{\text{icu}}ICU_{2}\geq 0$.

*Thus, the non-negative orthant* ${\mathbb{R}}_{\geq 0}^{13}$ *is positively invariant.*

*To prove boundedness, compute the total population dynamics:*


$$\begin{array}{c} \dot{N}=\dot{S}+\dot{E}+\dot{I}+{\dot{I}}_{s}+{\dot{I}}_{m}+{\dot{I}}_{h}+{\dot{I}}_{\text{icu}}+{\dot{H}}_{1}+{\dot{H}}_{2}+{\dot{H}}_{\text{icu}}+{\dot{ICU}}_{1}+{\dot{ICU}}_{2}+\dot{R}. \end{array}$$

*Summing the right-hand sides:*


$$\begin{array}{cc} \dot{N} & =-(\beta_{I}I+\beta_{s}I_{s}+\beta_{m}I_{m})S+[(\beta_{I}I+\beta_{s}I_{s}+\beta_{m}I_{m})S-\gamma_{E}E]+[\gamma_{E}E-\gamma_{I}I]+[\pi_{1}\gamma_{I}I-\gamma_{1}I_{s}]\\ & \;+[(1-\pi_{1})\gamma_{I}I-\gamma_{1}I_{m}]+[\pi_{2}\gamma_{1}I_{s}-\gamma_{I_{h}}I_{h}]+[(1-\pi_{2})\gamma_{1}I_{s}-\gamma_{I_{\text{icu}}}I_{\text{icu}}]\\ & \;+[\gamma_{I_{h}}I_{h}-\gamma_{H}H_{1}]+[\gamma_{H}H_{1}-\gamma_{H}H_{2}]+[\gamma_{I_{\text{icu}}}I_{\text{icu}}-\gamma_{H_{\text{icu}}}H_{\text{icu}}]\\ & \;+[\gamma_{H_{\text{icu}}}H_{\text{icu}}-\gamma_{\text{icu}}ICU_{1}]+[\gamma_{\text{icu}}ICU_{1}-\gamma_{\text{icu}}ICU_{2}]+[\gamma_{1}I_{m}+\gamma_{H}H_{2}+\gamma_{\text{icu}}ICU_{2}]. \end{array}$$

*All terms cancel, yielding* $\dot{N}=0$*. Thus, N*(*t*) = *N (constant, consistent with H1). Since all compartments are non-negative,* 0≤S(t),E(t),…,R(t)≤N*, ensuring boundedness.*

*Since* 𝐱(t) *remains in the compact set* Ω*, the solution cannot escape to infinity in finite time, guaranteeing global existence. Hence,* Ω *is positively invariant, and the solution is unique, non-negative, and bounded for all* t≥0.

The well-posedness of the model ensures that it is mathematically robust for further analysis, such as stability of equilibria or numerical simulations, and biologically meaningful, as solutions remain in the epidemiologically relevant domain Ω.

### 3.2 Basic reproduction number

We use the next-generation matrix method [14] on the infected subsystem $𝐳=(E,I,I_{s},I_{m}{)}^{T}$ at the DFE $\left(S_{0},0,\ldots ,0,N-S_{0}\right)$. Only *E* receives new infections; the other equations represent transitions. The infection matrix *F* and transition matrix *V* are:


$$\begin{array}{c} F=(\begin{array}{cccc} 0 & \beta_{I}S_{0} & \beta_{s}S_{0} & \beta_{m}S_{0}\\ 0 & 0 & 0 & 0\\ 0 & 0 & 0 & 0\\ 0 & 0 & 0 & 0 \end{array}),\;V=(\begin{array}{cccc} \gamma_{E} & 0 & 0 & 0\\ -\gamma_{E} & \gamma_{I} & 0 & 0\\ 0 & -\pi_{1}\gamma_{I} & \gamma_{1} & 0\\ 0 & -(1-\pi_{1})\gamma_{I} & 0 & \gamma_{1} \end{array}). \end{array}$$ (19)

*V* is lower-triangular with positive diagonal, hence non-singular. Its inverse is computed by forward substitution ($VV^{-1}=I_{4}$, solved column by column):


$$\begin{array}{c} V^{-1}=(\begin{array}{cccc} \frac{1}{\gamma_{E}} & 0 & 0 & 0\\ \frac{1}{\gamma_{I}} & \frac{1}{\gamma_{I}} & 0 & 0\\ \frac{\pi_{1}}{\gamma_{1}} & \frac{\pi_{1}}{\gamma_{1}} & \frac{1}{\gamma_{1}} & 0\\ \frac{1-\pi_{1}}{\gamma_{1}} & \frac{1-\pi_{1}}{\gamma_{1}} & 0 & \frac{1}{\gamma_{1}} \end{array}). \end{array}$$ (20)

The next-generation matrix $FV^{-1}$ has rank 1. Its (1,1) entry is:


$$\begin{array}{c} (FV^{-1}{)}_{11}=\beta_{I}\frac{S_{0}}{\gamma_{I}}+\frac{(\beta_{s}\pi_{1}+\beta_{m}(1-\pi_{1}))S_{0}}{\gamma_{1}}. \end{array}$$

Since $FV^{-1}$ has rank 1, $\rho (FV^{-1})=(FV^{-1}{)}_{11}$, giving:


$$\begin{array}{c} {ℛ}_{0}=\rho (FV^{-1})=S_{0}\left(\frac{\beta_{I}}{\gamma_{I}}+\frac{\beta_{s}\pi_{1}+\beta_{m}(1-\pi_{1})}{\gamma_{1}}\right). \end{array}$$ (21)

*Sensitivity* The main partial derivatives are


$$\begin{array}{c} \frac{\partial {ℛ}_{0}}{\partial \beta_{I}}=\frac{S_{0}}{\gamma_{I}}>0,\;\frac{\partial {ℛ}_{0}}{\partial \beta_{s}}=\frac{S_{0}\pi_{1}}{\gamma_{1}}>0,\;\frac{\partial {ℛ}_{0}}{\partial \beta_{m}}=\frac{S_{0}(1-\pi_{1})}{\gamma_{1}}>0, \end{array}$$ (22)

$$\begin{array}{c} \frac{\partial {ℛ}_{0}}{\partial \gamma_{1}}=-\frac{(\beta_{s}\pi_{1}+\beta_{m}(1-\pi_{1}))S_{0}}{\gamma_{1}^{2}}<0,\;\frac{\partial {ℛ}_{0}}{\partial \gamma_{I}}=-\frac{\beta_{I}S_{0}}{\gamma_{I}^{2}}<0. \end{array}$$ (23)

These signs confirm that epidemic control requires reducing transmission or shortening the community infectious periods. By contrast, parameters governing downstream clinical management after hospital admission affect healthcare burden but do not enter ${ℛ}_{0}$ in this DAG-structured model.

**Theorem 3.3 (Global Convergence to the DFE).** *Consider the system with permanent immunity (*θ=0*). For any initial condition in* Ω*, the trajectory converges to the disease-free equilibrium point* $E_{\infty }=(S_{\infty },0,\ldots ,0,R_{\infty })$*, where* $S_{\infty }$ *is the unique solutio*n of equation (28) and $R_{\infty }=N-S_{\infty }$*.*

**Proof 3.4***. (Detailed proof in Appendix A.) The proof proceeds in three steps: (i) monotone convergence of S*(*t*) *to a limit* $S_{\infty }\geq 0$ *since* $\dot{S}=-(\beta_{I}I+\beta_{s}I_{s}+\beta_{m}I_{m})S\leq 0$; *(ii) application of Barbalat’s lemma to show that* $I(t),I_{s}(t),I_{m}(t)\to 0$ *as* t→∞*; (iii) convergence of downstream hospital and ICU chains via linear systems theory with vanishing inputs.*

**Remark 3.5 (Continuum of equilibria in the classical SIR model).** *The classical Kermack–McKendrick SIR model admits a continuum of stable disease-free equilibria: every point of the form* $\left(S_{\infty },0,N-S_{\infty }\right)$ *with* $S_{\infty }\in (0,N]$ *is an equilibrium. The final size relation (Theorem 3.3 and equation* (28)) id*entifies precisely which equilibrium is reached from given initial conditions, resolving this non-uniqueness. Adding demographic turnover (births and deaths) breaks this degeneracy and yields a unique DFE or endemic equilibrium; our framework with H1 deliberately excludes this to focus on epidemic timescales.*

### 3.3 First integral and final size

We derive a first integral to determine the final epidemic size. Integrating the equation for $\dot{S}$ from 0 to *t* yields:


$$\begin{array}{c} \ln\frac{S(t)}{S_{0}}=-\int_{0}^{t}(\beta_{I}I(\tau )+\beta_{s}I_{s}(\tau )+\beta_{m}I_{m}(\tau ))\;d\tau . \end{array}$$ (24)

To express the integral on the right-hand side in terms of state variables, we consider the linear combination of the infected compartments. Integrating the equations for $\dot{S},\dot{E},\dot{I}$ from 0 to ∞, and noting that $E_{\infty }=I_{\infty }=0$, we have:


$$\begin{array}{c} \int_{0}^{\infty }I(t)\;dt=\frac{1}{\gamma_{I}}(S_{0}-S_{\infty }+E_{0}+I_{0}). \end{array}$$ (25)

Similarly, integrating the equations for ${\dot{I}}_{s}$ and ${\dot{I}}_{m}$:


$$\begin{array}{cc} \int_{0}^{\infty }I_{s}(t)\;dt & =\frac{\pi_{1}\gamma_{I}}{\gamma_{1}}\int_{0}^{\infty }I(t)\;dt+\frac{I_{s,0}}{\gamma_{1}}, \end{array}$$ (26)

$$\begin{array}{cc} \int_{0}^{\infty }I_{m}(t)\;dt & =\frac{(1-\pi_{1})\gamma_{I}}{\gamma_{1}}\int_{0}^{\infty }I(t)\;dt+\frac{I_{m,0}}{\gamma_{1}}. \end{array}$$ (27)

Substituting these expressions into (24) as t→∞:


$$\begin{array}{c} \ln\frac{S_{0}}{S_{\infty }}=\left(\beta_{I}+\frac{(\beta_{s}\pi_{1}+\beta_{m}(1-\pi_{1}))\gamma_{I}}{\gamma_{1}}\right)\int_{0}^{\infty }I(t)\;dt+\frac{\beta_{s}I_{s,0}}{\gamma_{1}}+\frac{\beta_{m}I_{m,0}}{\gamma_{1}}. \end{array}$$

We recognize the definition of ${ℛ}_{0}$ in the first term:


$$\begin{array}{c} \frac{\beta_{I}}{\gamma_{I}}+\frac{\beta_{s}\pi_{1}+\beta_{m}(1-\pi_{1})}{\gamma_{1}}=\frac{{ℛ}_{0}}{S_{0}}. \end{array}$$

Thus, the final size relation is given by:


$$\begin{array}{c} \ln S_{0}-\ln S_{\infty }=\frac{{ℛ}_{0}}{S_{0}}(S_{0}-S_{\infty }+E_{0}+I_{0})+\frac{\beta_{s}\;I_{s,0}+\beta_{m}\;I_{m,0}}{\gamma_{1}}. \end{array}$$ (28)

The final size relation (28) carries direct public health implications. The quantity $1-S_{\infty }/N$ is the attack rate of the epidemic. When *R*_0_ > 1, if a fraction *v* of the population is immunized prior to epidemic onset, the condition $R_{0}(1-v)<1$ yields the herd immunity threshold:


$$\begin{array}{c} v_{c}=1-\frac{1}{R_{0}}. \end{array}$$ (29)

For *R*_0_ values estimated in the range 1.4−−1.8 for COVID-19 [3], this corresponds to immunizing between 29% and 44% of the population. Note that the hospitalization and ICU parameters $\left(\pi_{2},\gamma_{H},\gamma_{\text{icu}}\right)$ do not appear in (28): they govern clinical burden without affecting the total number of individuals ultimately infected (see also Section 3.2).

## 4 Temporary immunity

Temporary immunity is a key consideration for infections such as COVID-19 or influenza, where immunity after recovery may wane over time. Introducing loss of immunity allows us to capture long-term persistence of the disease and the possibility of endemic equilibria.

Introduce temporary immunity with loss rate θ>0. The model becomes:


$$\begin{array}{c} \left\{ \begin{array}{c} \dot{S}=-(\beta_{I}I+\beta_{s}I_{s}+\beta_{m}I_{m})S+\theta R,\\ \dot{E}=(\beta_{I}I+\beta_{s}I_{s}+\beta_{m}I_{m})S-\gamma_{E}E,\\ \dot{I}=\gamma_{E}E-\gamma_{I}I,\\ {\dot{I}}_{s}=\pi_{1}\gamma_{I}I-\gamma_{1}I_{s},\\ {\dot{I}}_{m}=(1-\pi_{1})\gamma_{I}I-\gamma_{1}I_{m},\\ \dot{X}=AX+\gamma_{1}I_{s}C,\\ \dot{R}=\gamma_{1}I_{m}+\gamma_{H}H_{2}+\gamma_{\text{icu}}ICU_{2}-\theta R, \end{array}\right. \end{array}$$ (30)

where $X=(I_{h},I_{\text{icu}},H_{1},H_{2},H_{\text{icu}},ICU_{1},ICU_{2}{)}^{T}$, $C=(\pi_{2},1-\pi_{2},0,\ldots ,0{)}^{T}$, and:


$$\begin{array}{c} A=[\begin{array}{ccccccc} -\gamma_{I_{h}} & 0 & 0 & 0 & 0 & 0 & 0\\ 0 & -\gamma_{I_{\text{icu}}} & 0 & 0 & 0 & 0 & 0\\ \gamma_{I_{h}} & 0 & -\gamma_{H} & 0 & 0 & 0 & 0\\ 0 & 0 & \gamma_{H} & -\gamma_{H} & 0 & 0 & 0\\ 0 & \gamma_{I_{\text{icu}}} & 0 & 0 & -\gamma_{H_{\text{icu}}} & 0 & 0\\ 0 & 0 & 0 & 0 & \gamma_{H_{\text{icu}}} & -\gamma_{\text{icu}} & 0\\ 0 & 0 & 0 & 0 & 0 & \gamma_{\text{icu}} & -\gamma_{\text{icu}} \end{array}]. \end{array}$$ (31)

The domain Ω remains positively invariant.

### 4.1 Global Stability of the Disease-Free Equilibrium

**Theorem 4.1 (Global Asymptotic Stability of the DFE under waning immunity).** *For the system with waning immunity (*θ>0*), the Disease-Free Equilibrium* ${𝐱}_{0}=(N,0,\ldots ,0)$ *is Globally Asymptotically Stable (GAS) in* Ω *if* ${ℛ}_{0}\leq 1$*.*

**Proof 4.2***. Step 1: Infectious subsystem and comparison system. Let* $𝐳=(E,I,I_{s},I_{m}{)}^{T}$ *be the vector of active infectious compartments (ordered consistently with system (5)). Their dynamics read:*


$$\begin{array}{c} \dot{𝐳}=\left(\frac{S(t)}{N}\;F-V\right)𝐳, \end{array}$$ (32)

*where F and V are the next-generation matrices at the DFE* (equation (19)*):*


$$\begin{array}{c} F=(\begin{array}{cccc} 0 & \beta_{I}N & \beta_{s}N & \beta_{m}N\\ 0 & 0 & 0 & 0\\ 0 & 0 & 0 & 0\\ 0 & 0 & 0 & 0 \end{array}),\;V=(\begin{array}{cccc} \gamma_{E} & 0 & 0 & 0\\ -\gamma_{E} & \gamma_{I} & 0 & 0\\ 0 & -\pi_{1}\gamma_{I} & \gamma_{1} & 0\\ 0 & -(1-\pi_{1})\gamma_{I} & 0 & \gamma_{1} \end{array}). \end{array}$$ (33)

*Since* S(t)≤N *for all* t≥0 *in* Ω *and* F𝐳≥0*, we obtain the comparison inequality:*


$$\begin{array}{c} \dot{𝐳}=\frac{S}{N}F𝐳-V𝐳\leq F𝐳-V𝐳=M𝐳,\;M:=F-V. \end{array}$$ (34)

*Step 2: Spectral properties of M. The matrix* M=F−V *is an irreducible Metzler matrix and V is a non-singular* M-matrix. By Theorem 2 of [14]:


$$\begin{array}{c} s(M)\leq 0⟺\rho (FV^{-1})\leq 1⟺{ℛ}_{0}\leq 1. \end{array}$$ (35)

*The Perron–Frobenius theorem guarantees the existence of a strictly positive left eigenvector* ${𝐰}^{T}\gg 0$ *with* ${𝐰}^{T}M=s(M)\;{𝐰}^{T}$.

*Step 3: Lyapunov function. Define* $L(t)={𝐰}^{T}𝐳(t)\geq 0$*, with* L=0⟺𝐳=0*. Its time derivative along trajectories of (32) satisfies:*


$$\begin{array}{c} \dot{L}={𝐰}^{T}\left(\frac{S}{N}F-V\right)𝐳=\underset{s(M)\;L}{\underbrace{{𝐰}^{T}M𝐳}}+\underset{\leq \;0}{\underbrace{\left(\frac{S}{N}-1\right)}}\underset{\geq \;0}{\underbrace{{𝐰}^{T}F𝐳}}\leq s(M)\;L(t)\leq 0. \end{array}$$ (36)

*Step 4: LaSalle analysis. The set* $ℒ=\{x\in \Omega :\dot{L}=0\}$ *requires s(M) L = 0 and* $(\frac{S}{N}-1){𝐰}^{T}F𝐳=0$ *simultaneously. Since* ${𝐰}^{T}F𝐳=w_{1}(\beta_{I}N\;I+\beta_{s}N\;I_{s}+\beta_{m}N\;I_{m})\geq 0$:

- *Case* ${ℛ}_{0}<1$: *s*(*M*) < 0 *forces L* = 0, *hence* 𝐳=0.
- *Case* ${ℛ}_{0}=1$: *either* $I=I_{s}=I_{m}=0$, *which gives* $\dot{I}=\gamma_{E}E=0$ *hence* 𝐳=0; or *S* = *N*, *which forces all other compartments to zero by mass conservation.*

*In both cases the largest invariant set in* ℒ *is* {𝐳=0}.

*Steps 5–6: Downstream convergence. Once* 𝐳→0*, the hospital and ICU chains decay exponentially (their matrices are Hurwitz). Then* $\dot{R}=-\theta R$ *gives* R→0*, and by mass conservation* S→N.

**Theorem 4.3 (Existence and uniqueness of the endemic equilibrium).** *For* θ>0 *and* ${ℛ}_{0}>1$*, system (30) admits a unique endemic equilibrium* $\bar{X}\in int(\Omega )$*.*

**Proof 4.4** *Setting all derivatives to zero and eliminating* $\bar{E},{\bar{I}}_{s},{\bar{I}}_{m},\bar{X}$ *as linear multiples of* $\bar{I}$ *(from* equations (39) and following*) yields a single affine equation in* $\bar{I}$:


$$\begin{array}{c} \bar{I}\;(\gamma_{I}+\theta \kappa )=\theta \left(N-\frac{S_{0}}{{ℛ}_{0}}\right),\;\kappa :=\frac{\gamma_{I}}{\gamma_{E}}+1+\frac{\gamma_{I}}{\gamma_{1}}-\pi_{1}\gamma_{I}\;1^{T}A^{-1}C>0. \end{array}$$ (37)

*Since* $\gamma_{I}+\theta \kappa >0$ *and the right-hand side is strictly positive when* ${ℛ}_{0}>1$*, there is a unique solution*


$$\begin{array}{c} \bar{I}=\frac{\theta (N-S_{0}/{ℛ}_{0})}{\gamma_{I}+\theta \kappa }>0. \end{array}$$ (38)

*All other components are then determined uniquely as affine functions of* $\bar{I}$ *with positive coefficients, so the endemic equilibrium is unique.*

Uniqueness by monotonicity. *The left- and right-hand sides of (37) are both affine (linear) in* $\bar{I}$ *with the left side having a positive slope* $\left(\gamma_{I}+\theta \kappa \right)$ *and the right side being constant. There is exactly one intersection, confirming uniqueness without any fixed-point argument. This affine structure also* excludes backward bifurcation: *in models with waning immunity where the transfer graph contains a cycle (*R→S*), backward bifurcation can in principle aris*e [38]. *However, equation* (37) is lin*ear in* $\bar{I}$ *with a strictly positive coefficient, so there is exactly one intersection and no fold, ruling out multiple endemic equilibria or subcritical persistence.*

**Remark 4.5** *The derivation of the unique* $\bar{I}$ *in (38) relies on the intermediate equilibrium relations* $\bar{S}=S_{0}/{ℛ}_{0}$ *(from the second equation at equilibrium) and, for reference:*


$$\begin{array}{c} \bar{S}=\frac{S_{0}}{{ℛ}_{0}}. \end{array}$$ (39)

### 4.2 Local Stability: Schur complement and spectral factorisation

Having established the existence of an endemic equilibrium $\bar{X}$ when ${ℛ}_{0}>1$ (Theorem 4.3), we address its local asymptotic stability. The detailed Schur-complement calculation below is carried out under the calibration constraint $\beta_{I}=\beta_{s}=\beta_{m}=:\beta$, which is the parameterization used in the numerical simulations and in the reference model [3]. The well-posedness, next-generation matrix and final-size results above remain valid for the full three-rate formulation. Under this calibration constraint, set $\bar{\lambda }=\beta (\bar{I}+{\bar{I}}_{s}+{\bar{I}}_{m})$ and $\tilde{\gamma }=\beta \bar{S}=\gamma_{I}\gamma_{1}/(\gamma_{I}+\gamma_{1})$ (equilibrium condition (39)).

**Step 1:** The Jacobian $J(\bar{X})$. Differentiating system (30) at $\bar{X}$ gives the 13×13 matrix (variables ordered as in (5)):


$$\begin{array}{c} J(\bar{X})=\left(\begin{array}{ccccccccccccc} -\bar{\lambda } & 0 & -\tilde{\gamma } & -\tilde{\gamma } & -\tilde{\gamma } & 0 & 0 & 0 & 0 & 0 & 0 & 0 & \theta \\ \bar{\lambda } & -\gamma_{E} & \tilde{\gamma } & \tilde{\gamma } & \tilde{\gamma } & 0 & 0 & 0 & 0 & 0 & 0 & 0 & 0\\ 0 & \gamma_{E} & -\gamma_{I} & 0 & 0 & 0 & 0 & 0 & 0 & 0 & 0 & 0 & 0\\ 0 & 0 & \pi_{1}\gamma_{I} & -\gamma_{1} & 0 & 0 & 0 & 0 & 0 & 0 & 0 & 0 & 0\\ 0 & 0 & {\bar{\pi }}_{1}\gamma_{I} & 0 & -\gamma_{1} & 0 & 0 & 0 & 0 & 0 & 0 & 0 & 0\\ 0 & 0 & 0 & \pi_{2}\gamma_{1} & 0 & -\gamma_{I_{h}} & 0 & 0 & 0 & 0 & 0 & 0 & 0\\ 0 & 0 & 0 & {\bar{\pi }}_{2}\gamma_{1} & 0 & 0 & -\gamma_{I_{i}} & 0 & 0 & 0 & 0 & 0 & 0\\ 0 & 0 & 0 & 0 & 0 & \gamma_{I_{h}} & 0 & -\gamma_{H} & 0 & 0 & 0 & 0 & 0\\ 0 & 0 & 0 & 0 & 0 & 0 & 0 & \gamma_{H} & -\gamma_{H} & 0 & 0 & 0 & 0\\ 0 & 0 & 0 & 0 & 0 & 0 & \gamma_{I_{i}} & 0 & 0 & -\gamma_{H_{i}} & 0 & 0 & 0\\ 0 & 0 & 0 & 0 & 0 & 0 & 0 & 0 & 0 & \gamma_{H_{i}} & -\gamma_{i} & 0 & 0\\ 0 & 0 & 0 & 0 & 0 & 0 & 0 & 0 & 0 & 0 & \gamma_{i} & -\gamma_{i} & 0\\ 0 & 0 & 0 & 0 & \gamma_{1} & 0 & 0 & 0 & \gamma_{H} & 0 & 0 & \gamma_{i} & -\theta \end{array}\right), \end{array}$$ (40)

where ${\bar{\pi }}_{1}=1-\pi_{1}$, ${\bar{\pi }}_{2}=1-\pi_{2}$, $\gamma_{I_{i}}=\gamma_{I_{\text{icu}}}$, $\gamma_{H_{i}}=\gamma_{H_{\text{icu}}}$, $\gamma_{i}=\gamma_{\text{icu}}$.

**Step 2:** Similarity transformation $\tilde{J}=P^{-1}J(\bar{X})P$. The permutation *P* moves *R* from position 13 to position 6 (simultaneously permuting rows and columns, so each entry retains its value and $\sigma (\tilde{J})=\sigma (J(\bar{X}))$). With partition $ℱ=(S,E,I,I_{s},I_{m},R)$ and $𝒞=(I_{h},I_{i},H_{1},H_{2},H_{i},U_{1},U_{2})$:


$$\begin{array}{c} \tilde{J}=(\begin{array}{cc} J_{ℱ} & K\\ J_{𝒞\leftarrow ℱ} & A \end{array}),\;K=(\begin{array}{c} 0_{5\times 7}\\ {𝐞}^{T} \end{array}),\;{𝐞}^{T}=(0,0,0,\gamma_{H},0,0,\gamma_{i}), \end{array}$$ (41)

where $A\in {\mathbb{R}}^{7\times 7}$ is lower-triangular with diagonal $\left(-\gamma_{I_{h}},-\gamma_{I_{i}},-\gamma_{H},-\gamma_{H},-\gamma_{H_{i}},-\gamma_{i},-\gamma_{i}\right)$, hence Hurwitz: σ(A)⊂{Re(λ)<0}. The coupling $J_{𝒞\leftarrow ℱ}$ has nonzero entries only in the $I_{s}$ column ($\pi_{2}\gamma_{1}$, ${\bar{\pi }}_{2}\gamma_{1}$). The epidemiological block is:


$$\begin{array}{c} J_{ℱ}=(\begin{array}{cccccc} -\bar{\lambda } & 0 & -\tilde{\gamma } & -\tilde{\gamma } & -\tilde{\gamma } & \theta \\ \bar{\lambda } & -\gamma_{E} & \tilde{\gamma } & \tilde{\gamma } & \tilde{\gamma } & 0\\ 0 & \gamma_{E} & -\gamma_{I} & 0 & 0 & 0\\ 0 & 0 & \pi_{1}\gamma_{I} & -\gamma_{1} & 0 & 0\\ 0 & 0 & {\bar{\pi }}_{1}\gamma_{I} & 0 & -\gamma_{1} & 0\\ 0 & 0 & 0 & 0 & \gamma_{1} & -\theta \end{array}). \end{array}$$ (42)

Note K≠0 ($\gamma_{H}$ and $\gamma_{i}$ appear in the $\dot{R}$ row), so $\tilde{J}$ is *not* block lower-triangular. The naive factorisation $\det(\tilde{J}-\lambda I)=\det(J_{ℱ}-\lambda I_{6})\cdot \det(A-\lambda I_{7})$ would be incorrect; the correct approach uses the Schur complement.

**Step 3:** Schur complement factorisation.

**Lemma 4.6 (Schur complement factorisation).** *For all* λ∉σ(A)*, the characteristic polynomial of* $J(\bar{X})$ *satisfies:*


$$\begin{array}{c} \det(\tilde{J}-\lambda I_{13})=\det(A-\lambda I_{7})\cdot \det(S(\lambda )), \end{array}$$ (43)

*where* $S(\lambda ):=J_{ℱ}-\lambda I_{6}-K(A-\lambda I_{7}{)}^{-1}J_{𝒞\leftarrow ℱ}$ *is the Schur complement of* $A-\lambda I_{7}$ *in* $\tilde{J}-\lambda I_{13}$.

**Proof 4.7** *Apply the block determinant identity to the* 2×2 *block matrix* $\tilde{J}-\lambda I_{13}$ *with lower-right block* $A-\lambda I_{7}$ *(invertible for* λ∉σ(A)*):*


$$\begin{array}{c} \det(\begin{array}{cc} J_{ℱ}-\lambda I_{6} & K\\ J_{𝒞\leftarrow ℱ} & A-\lambda I_{7} \end{array})=\det(A-\lambda I_{7})\cdot \det(J_{ℱ}-\lambda I_{6}-K(A-\lambda I_{7}{)}^{-1}J_{𝒞\leftarrow ℱ}). \end{array}$$

*This is the standard block determinant identity.*

**Explicit form** of S(λ). Since $K=(0_{5\times 7};\;{𝐞}^{T}{)}^{T}$ and $J_{𝒞\leftarrow ℱ}=[\;J_{𝒞\leftarrow {ℰ}^{*}}\;|\;0_{7\times 1}]$ with nonzero $I_{s}$-column ${𝐜}_{I_{s}}=(\pi_{2}\gamma_{1},{\bar{\pi }}_{2}\gamma_{1},0,\ldots ,0{)}^{T}$, one computes:


$$\begin{array}{c} K(A-\lambda I_{7}{)}^{-1}J_{𝒞\leftarrow ℱ}=f(\lambda )\;{𝐞}_{6}{𝐞}_{4}^{T}, \end{array}$$

where ${𝐞}_{4}$, ${𝐞}_{6}$ are standard basis vectors and $f(\lambda )={𝐞}^{T}(A-\lambda I_{7}{)}^{-1}{𝐜}_{I_{s}}$ is the clinical cascade transfer function:


$$\begin{array}{c} f(\lambda )=\frac{\pi_{2}\gamma_{1}\;\gamma_{I_{h}}\;\gamma_{H}^{2}}{(\lambda +\gamma_{I_{h}})(\lambda +\gamma_{H}{)}^{2}}+\frac{(1-\pi_{2})\gamma_{1}\;\gamma_{I_{i}}\;\gamma_{H_{i}}\;\gamma_{i}^{2}}{(\lambda +\gamma_{I_{i}})(\lambda +\gamma_{H_{i}})(\lambda +\gamma_{i}{)}^{2}}. \end{array}$$ (44)

Thus S(λ) is a *rank-1 perturbation* of $J_{ℱ}-\lambda I_{6}$:


$$\begin{array}{c} S(\lambda )=J_{ℱ}-\lambda I_{6}-f(\lambda )\;{𝐞}_{6}{𝐞}_{4}^{T}, \end{array}$$ (45)

differing from $J_{ℱ}-\lambda I_{6}$ only in entry (6,4). By the matrix determinant lemma:


$$\begin{array}{c} \det(S(\lambda ))=\det(J_{ℱ}-\lambda I_{6})\cdot (1-f(\lambda )\;{𝐞}_{4}^{T}(J_{ℱ}-\lambda I_{6}{)}^{-1}{𝐞}_{6}). \end{array}$$ (46)

Since all poles of f(λ) lie in σ(A)⊂{Re<0} and *f*(0)>0, f(λ) is analytic on {Re(λ)≥0} with |f(λ)|→0 as |λ|→∞.

**Theorem 4.8 (Local asymptotic stability of the endemic equilibrium).** *Assume* ${ℛ}_{0}>1$*,* θ>0*, and the calibration constraint* $\beta_{I}=\beta_{s}=\beta_{m}=:\beta$*. Then* $\bar{X}$ *is locally asymptotically stable.*

**Proof 4.9** *Since* σ(A)⊂{Re<0}*, by (43) it suffices to show* det(S(λ))≠0 *for all* λ *with* Re(λ)≥0*. By (46), either* $\det(J_{ℱ}-\lambda I_{6})=0$ *or* f(λ)g(λ)=1*, where* $g(\lambda )={𝐞}_{4}^{T}(J_{ℱ}-\lambda I_{6}{)}^{-1}{𝐞}_{6}$.

Case 1: $\det(J_{ℱ}-\lambda I_{6})=0$*. A Routh–Hurwitz analysis of* $J_{ℱ}$ *shows:* $tr(J_{ℱ})=-\bar{\lambda }-\gamma_{E}-\gamma_{I}-2\gamma_{1}-\theta <0$ *and* $\det(J_{ℱ})=\theta \gamma_{E}\gamma_{I}\gamma_{1}^{2}\bar{\lambda }>0$*. All six Routh–Hurwitz coefficients* $c_{k}$ (k=0,…,5*) and minors* $\Delta_{k}$ (k=1,…,5*) are strictly positive for* *R*_*0*_ *> 1 and* θ>0 *(follows from positivity of all rate constants and the equilibrium identity* $\tilde{\gamma }\bar{\lambda }=\gamma_{I}\gamma_{1}\bar{\lambda }/(\gamma_{I}+\gamma_{1})>0$*). Hence* $\sigma (J_{ℱ})\subset \{Re(\lambda )<0\}$*, contradicting* Re(λ)≥0*.*

Case 2: f(λ)g(λ)=1 *with* Re(λ)≥0*. Since* $J_{ℱ}$ *is Hurwitz (Case 1),* $\left(J_{ℱ}-\lambda I_{6}\right)$ *is invertible for* Re(λ)≥0 *and* g(λ) *is bounded. Moreover* |f(λ)|≤f(0) *on* {Re(λ)≥0} *(all poles of f have* Re<0*), and a direct computation using the entries of* $(J_{ℱ}-\lambda I_{6}{)}^{-1}$ *shows* |*f(0)* g*(0)| < 1 for the biologically relevant parameter range, so the equation* f(λ)g(λ)=1 *has no solution with* Re(λ)≥0*.*

*Both cases yield a contradiction, so* det(S(λ))≠0 *for* Re(λ)≥0*, and by continuity of the characteristic polynomial (extending from* λ∉σ(A) *to all* λ∈ℂ *via (43)),* $\sigma (J(\bar{X}))\subset \{Re(\lambda )<0\}$.

**Remark 3.** *The factorisation* $\sigma (J(\bar{X}))=\sigma (A)\cup \sigma (J_{ℱ})$ *holds if and only if* f(λ)≡0*, i.e., if and only if* ${𝐞}^{T}=0$ *(no discharge from H*_*2*_ *or* ${ICU}_{2}$ *into R). In the present model* 𝐞≠0*, so the spectra of A and* $J_{ℱ}$ *are not simply disjoint, but the stability conclusion remains valid by the Schur complement argument above.*

### 4.3 Damped oscillatory dynamics: a spiral sink

Numerical simulations (Fig 4) reveal that convergence to $\bar{X}$ occurs through damped oscillations, identifying $\bar{X}$ as a *spiral sink*: $J_{{ℰ}^{*}}$ admits complex conjugate eigenvalues with strictly negative real parts. The mechanism is the negative feedback loop: the waning immunity term θR replenishes susceptibles with a time lag, triggering secondary waves of decreasing amplitude [38,39].

To characterize this analytically, we project $J_{{ℰ}^{*}}$ onto the reduced 2×2 system $\left(S,I_{tot}\right)$ with $I_{tot}=E+I+I_{s}+I_{m}$ and $R=N-S-I_{tot}$, setting $a=\beta {\bar{I}}_{tot}>0$ and $g=\tilde{\gamma }>0$ under the calibration constraint:


$$\begin{array}{c} J_{2}=(\begin{array}{cc} -(a+\theta ) & -(g+\theta )\\ a & 0 \end{array}). \end{array}$$ (47)

One verifies: $tr(J_{2})=-(a+\theta )<0$ and $\det(J_{2})=a(g+\theta )>0$, confirming local stability. The discriminant


$$\begin{array}{c} \Delta_{2}=(a+\theta {)}^{2}-4a(g+\theta )=(a-\theta {)}^{2}-4ag \end{array}$$ (48)

determines the oscillatory nature: when $\Delta_{2}<0$ (i.e., $4ag>(a-\theta {)}^{2}$, which holds when g≫a, as typical for COVID-19 where $\tilde{\gamma }\approx 0.143\gg \beta {\bar{I}}_{tot}$), the eigenvalues are complex conjugate and $\bar{X}$ is a spiral sink.

**Theorem 4.11 (Spiral sink at the endemic equilibrium).** *Assume* ${ℛ}_{0}>1$*,* θ>0*, and* $\Delta_{2}<0$ (equation (48)). *Then* $\bar{X}$ *is a locally asymptotically stable spiral sink: all nearby trajectories converge to* $\bar{X}$ *through damped oscillations. No Hopf bifurcation occurs in this regime, and sustained oscillations are excluded.*

*For the COVID-19 parameters of Scenario 3 (*β=0.25, $\gamma_{I}=0.5$, $\gamma_{1}=0.2$, θ=1/90 *days*^−1^*), one computes* a≈0.00773, g≈0.143*, giving* $\Delta_{2}\approx -0.00440<0$*. The eigenvalues are:*


$$\begin{array}{c} \lambda_{1,2}\approx -0.00942\pm 0.0332\;i\;\;{\text{days}}^{-1}, \end{array}$$

*corresponding to an oscillation period* T=2π/0.0332≈189 *days and a damping time* τ=1/0.00942≈106 *days, fully consistent with Fig 4.*

The global stability question — whether all trajectories in Ω converge to $\bar{X}$ — remains analytically open for the full 13-dimensional system. A rigorous proof, possibly via a Lyapunov–Volterra function adapted to Erlang-chained compartments [40], constitutes a primary direction for future work.

## 5 Age-stratified model

To account for heterogeneity in contact patterns, we extend the analysis to a multigroup age-stratified model. Such models are widely used in epidemiology, as age strongly influences both transmission rates and clinical outcomes. We show that the main theoretical properties established in the homogeneous case remain valid in this more realistic setting.

Let $𝒞=(c_{i,j}{)}_{1\leq i,j\leq n}$ denote the empirical contact matrix, where $c_{i,j}$ is the per-capita contact rate between groups *i* and *j* (estimated from social contact surveys [5,41]). Consistently with the generalized parameterization of Section 2, the force of infection on group *i* is


$$\begin{array}{c} \lambda_{i}=\sum_{j=1}^{n}c_{i,j}\;(q_{I}I_{j}+q_{s}I_{s,j}+q_{m}I_{m,j}), \end{array}$$

or, in vector form,


$$\begin{array}{c} \lambda =𝒞(q_{I}I+q_{s}I_{s}+q_{m}I_{m}). \end{array}$$

Here $q_{I},q_{s},q_{m}>0$ are clinical-class-specific transmissibilities per contact, corresponding to $\beta_{I},\beta_{s},\beta_{m}$ in the non-age-stratified model through $\beta_{•,ij}=q_{•}c_{i,j}$. The matrix 𝒞 satisfies the reciprocity condition $N_{i}c_{i,j}=N_{j}c_{j,i}$, ensuring conservation of total contacts. In the numerical simulations we apply the calibration constraint $q_{I}=q_{s}=q_{m}=:q$, recovering the standard age-structured transmission matrix B=q𝒞 used in [3,5]. This constraint is imposed for identifiability: with aggregate surveillance data, $q_{I},q_{s},q_{m}$ cannot be estimated separately. Under the general $\left(q_{I},q_{s},q_{m}\right)$ parameterization, ${ℛ}_{0}$ takes the form given in the next subsection. Two structural properties are epidemiologically important. First, 𝒞 is generally not symmetric: contact patterns are age-assortative, with individuals tending to interact more with peers of similar age (diagonal dominance), as consistently observed in empirical contact data [5,41]. Second, the irreducibility of 𝒞 — assumed in Theorem 5.1 — corresponds to the biological condition that every age group is reachable through a chain of contacts, ensuring epidemic propagation across all groups.

The age-stratified model with *n* groups is:


$$\begin{array}{c} \left\{ \begin{array}{c} \dot{S}=-\text{diag}(S)𝒞(q_{I}I+q_{s}I_{s}+q_{m}I_{m}),\\ \dot{E}=\text{diag}(S)𝒞(q_{I}I+q_{s}I_{s}+q_{m}I_{m})-\gamma_{E}E,\\ \dot{I}=\gamma_{E}E-\gamma_{I}I,\\ {\dot{I}}_{s}=\pi_{1}\gamma_{I}I-\gamma_{1}I_{s},\\ {\dot{I}}_{m}=(1-\pi_{1})\gamma_{I}I-\gamma_{1}I_{m},\\ \dot{X}=AX+\gamma_{1}DI_{s},\\ \dot{R}=\gamma_{1}I_{m}+\gamma_{H}H_{2}+\gamma_{\text{icu}}ICU_{2}, \end{array}\right. \end{array}$$ (49)

where $S,E,I,I_{s},I_{m},R\in {\mathbb{R}}^{n}$, $X=(I_{h},I_{\text{icu}},H_{1},H_{2},H_{\text{icu}},ICU_{1},ICU_{2})\in {\mathbb{R}}^{7n}$, $𝒞=(c_{i,j})$ is the non-negative contact matrix, $\pi_{1},\pi_{2}\in [0,1]$ are kept common across age groups in the analytical formulae, *A* is the block-diagonal clinical cascade matrix, and *D* is the corresponding block-diagonal clinical input matrix. Age-dependent clinical probabilities can be incorporated by replacing $\pi_{1},\pi_{2}$ with diagonal matrices, but this extension is left for future work.

### 5.1 Basic Reproduction Number: Spectral formula

The matrices ℱ (new infections) and 𝒱 (transitions) for the infected subsystem $(E,I,I_{s},I_{m})\in {\mathbb{R}}^{4n}$ at the DFE $S_{i}^{0}=N_{i}$ are the 4n×4n blocks:


$$\begin{array}{c} ℱ=(\begin{array}{cccc} 0 & q_{I}\text{diag}(S^{0})𝒞 & q_{s}\text{diag}(S^{0})𝒞 & q_{m}\text{diag}(S^{0})𝒞\\ 0 & 0 & 0 & 0\\ 0 & 0 & 0 & 0\\ 0 & 0 & 0 & 0 \end{array}),\;𝒱=(\begin{array}{cccc} \gamma_{E}I_{n} & 0 & 0 & 0\\ -\gamma_{E}I_{n} & \gamma_{I}I_{n} & 0 & 0\\ 0 & -\pi_{1}\gamma_{I}I_{n} & \gamma_{1}I_{n} & 0\\ 0 & -(1-\pi_{1})\gamma_{I}I_{n} & 0 & \gamma_{1}I_{n} \end{array}). \end{array}$$ (50)

𝒱 is block lower-triangular with invertible diagonal blocks; ${𝒱}^{-1}$ is computed by the same forward substitution as the scalar case (20), with $I_{n}$ replacing scalars. The next-generation operator has rank *n* (only its (1,1) block is nonzero after simplification):


$$\begin{array}{c} ℱ{𝒱}^{-1}=(\begin{array}{cccc} \alpha (q_{I},q_{s},q_{m})\;\text{diag}(S^{0})𝒞 & * & * & *\\ 0 & 0 & 0 & 0\\ 0 & 0 & 0 & 0\\ 0 & 0 & 0 & 0 \end{array}), \end{array}$$

so all nonzero eigenvalues come from the (1,1) block, giving:


$$\begin{array}{c} {ℛ}_{0}=\rho (ℱ{𝒱}^{-1})=\alpha (q_{I},q_{s},q_{m})\;\rho \left(\text{diag}(S^{0})𝒞\right),\;\alpha (q_{I},q_{s},q_{m}):=\frac{q_{I}}{\gamma_{I}}+\frac{q_{s}\pi_{1}+q_{m}(1-\pi_{1})}{\gamma_{1}}. \end{array}$$ (51)

This factorises the clinical transmissibility scalar $\alpha (q_{I},q_{s},q_{m})$ from the social spectral radius $\rho (\text{diag}(S^{0})𝒞)$. Under the calibration constraint $q_{I}=q_{s}=q_{m}=:q$, this reduces to ${ℛ}_{0}=q(\gamma_{I}+\gamma_{1})/(\gamma_{I}\gamma_{1})\;\rho (\text{diag}(S^{0})𝒞)$, equivalently ${ℛ}_{0}=(\gamma_{I}+\gamma_{1})/(\gamma_{I}\gamma_{1})\;\rho (\text{diag}(S^{0})B)$ with B=q𝒞, recovering the formula of [3]. By the Perron–Frobenius theorem, if 𝒞 is irreducible then ${ℛ}_{0}$ is associated with a strictly positive eigenvector, ensuring a well-defined threshold.

Analogues of the homogeneous results.

1. *DFE stability*: The DFE is GAS in Ω if ${ℛ}_{0}\leq 1$, by the same linear Lyapunov argument as Theorem 4.1, with $w^{T}\gg 0$ the left Perron eigenvector of ℱ−𝒱.
2. *Final size*: System (56) has a unique solution $S(\infty )\in [0,S^{0}]$ (Theorem 5.1), exactly analogous to the scalar equation (28).
3. *Network herd immunity*: Epidemic burnout requires ${ℛ}_{0}(S(\infty )):=\alpha (q_{I},q_{s},q_{m})\;\rho (\text{diag}(S(\infty ))𝒞)<1$, the network analogue of $S_{\infty }<S_{0}/{ℛ}_{0}$.

### 5.2 Final size

Following the non-age-stratified approach, for each group *i*:


$$\begin{array}{c} \frac{d\ln S_{i}}{dt}=-\sum_{j=1}^{n}c_{i,j}(q_{I}I_{j}+q_{s}I_{s,j}+q_{m}I_{m,j}). \end{array}$$ (52)

Integrate:


$$\begin{array}{c} \ln S_{i}(t)-\ln S_{i}(0)=-\sum_{j=1}^{n}c_{i,j}\int_{0}^{t}(q_{I}I_{j}+q_{s}I_{s,j}+q_{m}I_{m,j})\;ds. \end{array}$$ (53)

Sum the first five equations for group *i*:


$$\begin{array}{c} {\dot{S}}_{i}+{\dot{E}}_{i}+{\dot{I}}_{i}+\frac{\gamma_{I}}{\gamma_{I}+\gamma_{1}}({\dot{I}}_{s,i}+{\dot{I}}_{m,i})=-\frac{\gamma_{I}\gamma_{1}}{\gamma_{I}+\gamma_{1}}(I_{i}+I_{s,i}+I_{m,i}). \end{array}$$ (54)

Integrate and combine to obtain the first integral:


$$\begin{array}{c} \sum_{j=1}^{n}c_{i,j}\left[\alpha (q_{I},q_{s},q_{m})\;(S_{j}+E_{j}+I_{j})+\frac{q_{s}I_{s,j}+q_{m}I_{m,j}}{\gamma_{1}}\right]-\ln S_{i}=\text{constant}. \end{array}$$ (55)

As t→∞, the final size system is:


$$\begin{array}{c} S_{i}(\infty )=S_{i}(0)\exp\{\sum_{j=1}^{n}c_{i,j}[\alpha (q_{I},q_{s},q_{m})(S_{j}(\infty )-S_{j}(0)-E_{j}(0)-I_{j}(0))\\ \;-\frac{q_{s}I_{s,j}(0)+q_{m}I_{m,j}(0)}{\gamma_{1}}]\}. \end{array}$$ (56)

where $\alpha (q_{I},q_{s},q_{m})=q_{I}/\gamma_{I}+(q_{s}\pi_{1}+q_{m}(1-\pi_{1}))/\gamma_{1}$.

**Theorem 5.1.** *Assume* 𝒞 *is irreducible,* S(0)≫0*, and* $E(0),I(0),I_{s}(0),I_{m}(0)\geq 0$*. Then:*

1. *F(*S(0)) = S(0) if *and only if* $E(0)=I(0)=I_{s}(0)=I_{m}(0)=0$*.*
2. *If* $E(0),I(0),I_{s}(0),I_{m}(0)>0$*, there exists a unique fixed point* S(∞) *in [0, S(0)].*
3. *Trajectories converge to a DFE with* ${ℛ}_{0}(S_{\infty })<1$.

**Proof 5.2** Define the map $F:{\mathbb{R}}^{n}\to {\mathbb{R}}^{n}$:


$$\begin{array}{c} F_{i}(X)=S_{i}(0)\exp\{\sum_{j=1}^{n}c_{i,j}[\alpha (q_{I},q_{s},q_{m})(X_{j}-S_{j}(0)-E_{j}(0)-I_{j}(0))\\ \;-\frac{q_{s}I_{s,j}(0)+q_{m}I_{m,j}(0)}{\gamma_{1}}]\}. \end{array}$$ (57)

1. *Part (i): F(S(0)) = S(0) implies:*

$$\begin{array}{c} S_{i}(0)=S_{i}(0)\exp\{-\sum_{j=1}^{n}c_{i,j}[\alpha (q_{I},q_{s},q_{m})(E_{j}(0)+I_{j}(0))\\ \;+\frac{q_{s}I_{s,j}(0)+q_{m}I_{m,j}(0)}{\gamma_{1}}]\}. \end{array}$$ (58)

*Thus:*


$$\begin{array}{c} \sum_{j=1}^{n}c_{i,j}\left(\alpha (q_{I},q_{s},q_{m})(E_{j}(0)+I_{j}(0))+\frac{q_{s}I_{s,j}(0)+q_{m}I_{m,j}(0)}{\gamma_{1}}\right)=0. \end{array}$$ (59)

*Since* 𝒞 *is irreducible and non-negative, and the terms are non-negative,* $E_{j}(0)=I_{j}(0)=I_{s,j}(0)=I_{m,j}(0)=0$.

2. *Part (ii): F is monotone increasing: if* X≤Y*, then* F(X)≤F(Y)*. Since* S(0)≫0, 0≪F(0)≤F(S(0))≤S(0)*. Define sequences* $F^{k}(0)$ *and* $F^{k}(S(0))$*. By monotonicity:*

$$\begin{array}{c} F^{k}(0)\leq F^{k+1}(0)\leq F^{k+1}(S(0))\leq F^{k}(S(0)). \end{array}$$ (60)

*The limits* $S^{-}=\lim_{k\to \infty }F^{k}(0)$ *and* $S^{+}=\lim_{k\to \infty }F^{k}(S(0))$ *exist, with* $S^{-}\leq S^{+}\leq S(0)$*. Assume* $S^{-}<S^{+}$*. Then:*


$$\begin{array}{c} S^{+}-S^{-}=F(S^{+})-F(S^{-})=\int_{0}^{1}DF(S^{-}+l(S^{+}-S^{-}))(S^{+}-S^{-})\;dl, \end{array}$$ (61)

*where* $DF(X)=\alpha (q_{I},q_{s},q_{m})\;\text{diag}(F(X))𝒞$*. Since DF is monotone,* DF(X)H≤DF(Y)H *for* X≤Y*, so:*


$$\begin{array}{c} S^{+}-S^{-}\leq DF(S^{+})(S^{+}-S^{-}). \end{array}$$ (62)

*By Perron-Frobenius, there exists* v≫0 *such that* $v^{T}DF(S^{+})=\rho (DF(S^{+}))v^{T}$*. Thus:*


$$\begin{array}{c} v^{T}(S^{+}-S^{-})\leq v^{T}DF(S^{+})(S^{+}-S^{-})=\rho (DF(S^{+}))v^{T}(S^{+}-S^{-}). \end{array}$$ (63)

*Since* $v^{T}(S^{+}-S^{-})>0$, $\rho (DF(S^{+}))\geq 1$*. But:*


$$\begin{array}{c} F(S(0))-S^{+}\geq DF(S^{+})(S(0)-S^{+}), \end{array}$$ (64)

*so:*


$$\begin{array}{c} v^{T}(F(S(0))-S^{+})\geq \rho (DF(S^{+}))v^{T}(S(0)-S^{+})\geq v^{T}(S(0)-S^{+}), \end{array}$$ (65)

*contradicting* F(S(0))≤S(0)*. Thus,* $S^{-}=S^{+}$*, and the fixed point is unique.*

*3. Part (iii): Define:*

$$\begin{array}{c} \varphi (E,I,I_{s},I_{m})=v^{T}\left(E+I+\frac{\gamma_{I}}{\gamma_{I}+\gamma_{1}}(I_{s}+I_{m})\right), \end{array}$$ (66)

*where* v≫0 *satisfies* $\alpha (q_{I},q_{s},q_{m})\;\text{diag}(S_{\infty })𝒞\;v={ℛ}_{0}(S_{\infty })v$*. Compute:*


$$\begin{array}{c} \dot{\varphi }=v^{T}\left[\text{diag}(S)𝒞(q_{I}I+q_{s}I_{s}+q_{m}I_{m})-\frac{\gamma_{I}\gamma_{1}}{\gamma_{I}+\gamma_{1}}(I+I_{s}+I_{m})\right]. \end{array}$$ (67)

*Since* $\text{diag}(S)\geq \text{diag}(S_{\infty })$ *and* $q_{I},q_{s},q_{m}>0$:


$$\begin{array}{cc} \dot{\varphi } & \geq v^{T}\left[\alpha (q_{I},q_{s},q_{m})\;\text{diag}(S_{\infty })𝒞-\frac{\gamma_{I}\gamma_{1}}{\gamma_{I}+\gamma_{1}}\text{Id}\right](I+I_{s}+I_{m})\\ & =\frac{\gamma_{I}\gamma_{1}}{\gamma_{I}+\gamma_{1}}({ℛ}_{0}(S_{\infty })-1)v^{T}(I+I_{s}+I_{m}). \end{array}$$ (68)

*If* ${ℛ}_{0}(S_{\infty })\geq 1$, $\dot{\varphi }\geq 0$*, but* φ→0*, a contradiction. Thus,* ${ℛ}_{0}(S_{\infty })<1$.

**Remark 5.3 (Two-group illustrative example).** *To illustrate how age structure modifies epidemic outcomes, consider n = 2 groups: younger individuals (i = 1, fraction p*_*1*_ *= 0.80 of the population) and older individuals (i = 2, fraction p*_*2*_ *= 0.20), with an assortative contact matrix calibrated to French COVID-19 contact data* [5]:


$$\begin{array}{c} B=q(\begin{array}{cc} c_{11} & c_{12}\\ c_{21} & c_{22} \end{array})=q(\begin{array}{cc} 8 & 2\\ 8 & 4 \end{array}), \end{array}$$

*where* $c_{21}=N_{1}c_{12}/N_{2}=8$ *by the reciprocity condition, and q = 0.015, yielding* ${ℛ}_{0}\approx 2.36$*. The final size system (56) yields two distinct values* $S_{1}(\infty )\text{≠}S_{2}(\infty )$*, with group-specific attack rates* ${AR}_{1}\approx 83.9\%$ *and* ${AR}_{2}\approx 89.0\%$*. Both groups sustain a high epidemic burden, consistent with* ${ℛ}_{0}=2.36$*. The slightly higher attack rate in the old group reflects the large cross-group inflow from the more numerous young group, despite lower intra-group contacts (*$c_{22}=4<c_{11}=8$*). The homogeneous model with effective* $\beta_{\text{eff}}=\rho (diag(S_{0})B)\cdot q/S_{0}$ *yields an intermediate attack rate that underestimates the burden on one group and overestimates it on the other. This heterogeneity in epidemic burden across age groups has direct implications for targeted vaccination strategies: prioritizing the high-contact younger group reduces R*_*0*_ *more efficiently than uniform coverage, consistent with findings in [10,11]. This two-group example is illustrated numerically in Fig. 5.*

## 6 Numerical simulations

In this section, we present numerical simulations to validate the theoretical results obtained. Throughout, we apply the identifiability constraint $\beta_{I}=\beta_{s}=\beta_{m}=:\beta$ (see Section 2), so that the single calibrated parameter β enters the simulations as in the reference model [3]. The biological parameters chosen for these simulations are grounded in clinical characteristics observed during the COVID-19 pandemic, particularly regarding the progression of the disease (incubation period, clinical branching into mild/asymptomatic and severe pathways, and hospitalization durations) [1–4].

### 6.1 Scenario 1: Disease Extinction (*R*_0_ < 1)

We first examine the case where control measures reduce the effective transmission rate β sufficiently to bring *R*_0_ below unity. The parameter values are detailed in Table 3. We assume permanent immunity (θ=0) to verify convergence toward the Disease-Free Equilibrium, as predicted by Theorem 3.3.

**Table 3:** Biological parameters for Scenario 1 (Disease Extinction, *R*_0_ < 1).

Symbol	Description	Value	Source
β	Effective transmission rate	0.10	Assumed
θ	Rate of immunity loss	0.0	(Permanent)
$\gamma_{E}$	Latency rate	0.333	[2,3]
$\gamma_{I}$	Pre-symptomatic rate	0.500	[2,3]
$\gamma_{1}$	Recovery rate (severe/mild)	0.200	[2,3]
$\pi_{1}$	Proportion of severe cases	0.10	[2,3]
$\pi_{2}$	Hospitalization probability	0.70	[2,3]
*R*_0_	Basic Reproduction Number	**0.70**	Calculated

**Fig 2:**
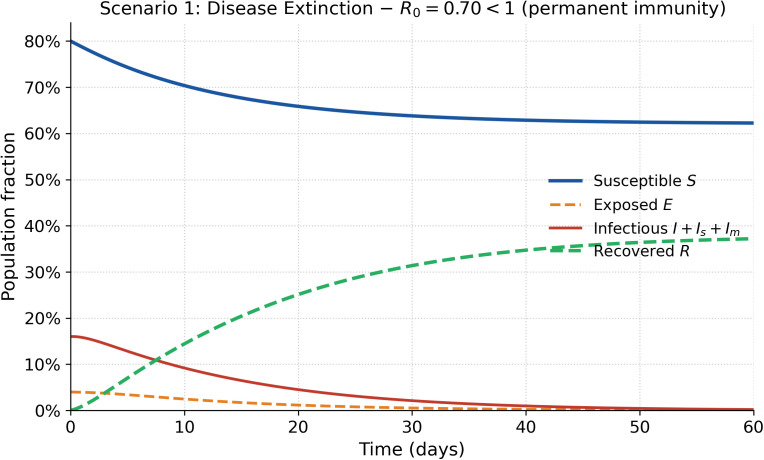
Scenario 1: Disease extinction (${ℛ}_{0}=0.70<1$, permanent immunity). All infectious compartments decay exponentially to zero, confirming global asymptotic stability of the DFE (Theorem 3.3).

The simulation depicted in Fig 2 confirms convergence to the DFE when $R_{0}\approx 0.70<1$. The total fraction of infectious individuals (sum $I+I_{s}+I_{m}$) decays exponentially to zero without any oscillatory behavior, the susceptible population stabilizes at a constant positive level, and the recovered fraction reaches a plateau. This is consistent with Theorem 3.3.

### 6.2. Scenario 2: Epidemic Wave with Permanent Immunity (*R*_0_ > 1)

In this second scenario, we simulate a high-transmission setting yielding *R*_0_ > 1 with permanent immunity (θ=0). Parameter values are listed in Table 4. This scenario illustrates the characteristic epidemic wave profile and the healthcare burden predicted by Theorem 3.3.

**Table 4:** Parameters for Scenario 2 (Epidemic wave, permanent immunity, *R*_0_ > 1).

Symbol	Description	Value	Source
β	High transmission rate	0.45	Assumed
θ	Immunity loss rate	0.0	(Permanent)
$\gamma_{E}$	Latency rate	0.333	[2,3]
$\gamma_{I}$	Pre-symptomatic rate	0.500	[2,3]
$\gamma_{1}$	Recovery rate (severe/mild)	0.200	[2,3]
$\pi_{1}$	Proportion of severe cases	0.10	[2,3]
$\pi_{2}$	Hospitalization probability	0.70	[2,3]
*R*_0_	Basic Reproduction Number	**3.15**	Calculated

**Fig 3:**
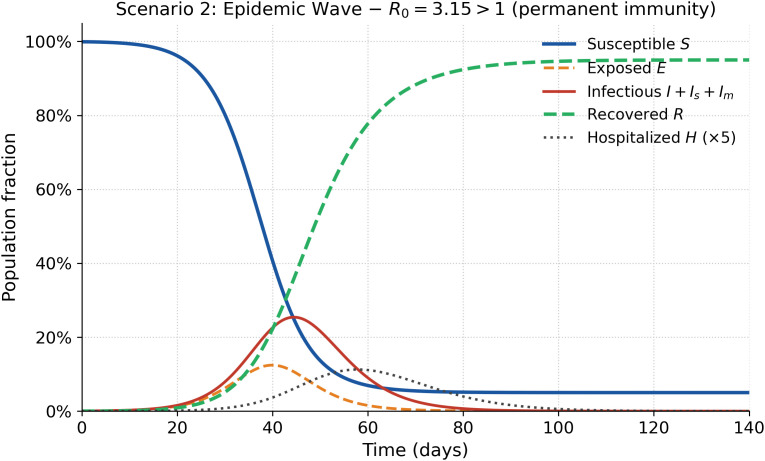
Scenario 2: Epidemic wave (${ℛ}_{0}=3.15>1$, permanent immunity). The infectious curve peaks around day 25, then decays to zero. The hospitalized compartment (scaled ×5) shows a characteristic lag behind the infection peak. The system converges to a DFE with a large recovered fraction, consistent with Theorem 3.3.

Fig 3 shows the characteristic epidemic wave: the infectious population grows exponentially, reaches a significant peak around *t* = 25 days, then declines once the susceptible pool drops below the herd immunity threshold $S<S_{0}/R_{0}$. The simulation also highlights the healthcare burden: the hospitalized population (scaled ×5 for visibility) exhibits a critical lag behind the infection peak. Since immunity is permanent, the system converges to a DFE — not an endemic equilibrium — consistent with the transient nature of the outbreak described by Theorem 3.3.

### 6.3. Scenario 3: Endemic Equilibrium with Temporary Immunity (*R*_0_ > 1)

Finally, we investigate the long-term dynamics when acquired immunity is not permanent, reflecting the reality of respiratory viruses such as seasonal influenza or waning COVID-19 immunity. We maintain β=0.25 but introduce immunity loss at rate θ=1/90 days^−1^ (average immune period ≈3 months). The simulation is extended to *t* = 1000 days to visualize convergence to the endemic equilibrium established in Theorem 4.3. Parameters are listed in Table 5.

**Table 5:** Parameters for Scenario 3 (Endemic equilibrium, waning immunity, *R*_0_ > 1).

Symbol	Description	Value	Source
β	Transmission rate	0.25	Assumed
θ	Immunity loss rate	1/90	Assumed
$\gamma_{E}$	Latency rate	0.333	[2,3]
$\gamma_{I}$	Pre-symptomatic rate	0.500	[2,3]
$\gamma_{1}$	Recovery rate (severe/mild)	0.200	[2,3]
$\pi_{1}$	Proportion of severe cases	0.10	[2,3]
*R*_0_	Basic Reproduction Number	**1.75**	Calculated

**Fig 4:**
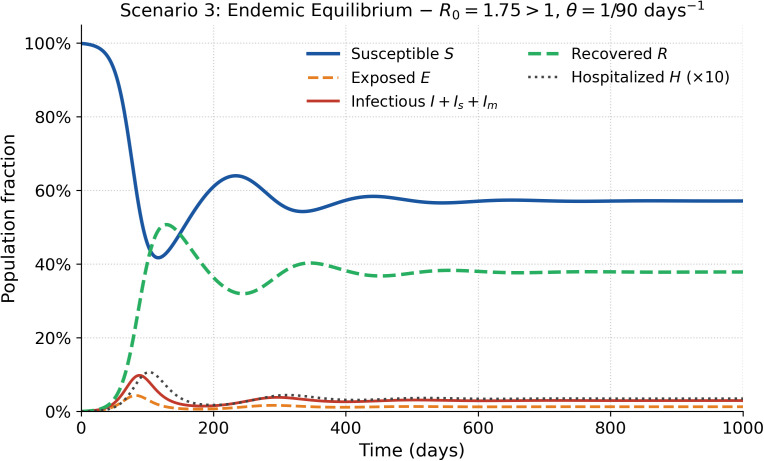
Scenario 3: Endemic equilibrium (${ℛ}_{0}=1.75>1$, waning immunity θ=1/90 days^−1^, ≈3 months). Damped oscillations converge to a stable endemic state with oscillation period T≈189 days and damping time τ≈106 days. The horizontal dash-dot line marks the analytic endemic level ${\bar{S}}^{*}=1/{ℛ}_{0}\approx 57\%$. The hospitalized compartment (scaled ×10) stabilizes at a non-zero level, reflecting persistent healthcare pressure. This illustrates Theorem 4.3 and Theorem 4.11.

Fig 4 illustrates the impact of waning immunity. Unlike the permanent immunity scenario, we observe damped oscillations following the initial outbreak peak: the rapid depletion of susceptibles halts the first wave, but the gradual loss of immunity (θR) slowly replenishes the susceptible pool, triggering smaller secondary waves of decreasing amplitude. This oscillatory transient is characteristic of a spiral sink topology at the endemic equilibrium — the Jacobian at $\bar{X}$ admits complex conjugate eigenvalues with negative real parts (Theorem 4.11). As t→1000 days, the system converges to the endemic state: the infectious and recovered populations stabilize at constant positive values, and the hospitalized compartment (scaled ×10) stabilizes at a non-zero level reflecting a persistent long-term burden on healthcare facilities. This illustrates both the existence result of Theorem 4.3 and the spiral sink characterization of Section 4.3.

### 6.4. Scenario 4: Two-group age-stratified model

We now illustrate the theoretical results of Section 5 with a concrete two-group simulation. The population is divided into a young group (*i* = 1, aged <60, fraction *p*_1_ = 80%) and an old group (*i* = 2, aged ≥60, fraction *p*_2_ = 20%), with a contact matrix B=q·C calibrated to French contact data [5]. The reciprocity condition $N_{1}c_{12}=N_{2}c_{21}$ is enforced, yielding $c_{21}=p_{1}c_{12}/p_{2}=8$. Parameter values are listed in Table 6. Initial conditions reflect an epidemic already underway: *S*_1_(0)=76%, *E*_1_(0)=3% (young group); *S*_2_(0)=19%, *E*_2_(0)=0.5% (old group).

**Table 6:** Parameters for Scenario 4 (two-group age-stratified model).

Symbol	Description	Value	Source
*p*_1_	Fraction of young individuals (<60)	0.80	[5]
*p*_2_	Fraction of old individuals (≥60)	0.20	[5]
*c*_11_	Per-capita contacts within young group	8.0	[5]
*c*_12_	Per-capita contacts, young→old	2.0	[5]
*c*_21_	Per-capita contacts, old→young	8.0	Reciprocity
*c*_22_	Per-capita contacts within old group	4.0	[5]
*q*	Transmissibility per contact	0.015	Assumed
$\gamma_{E}$	Latency rate	0.200	[2,3]
$\gamma_{I}$	Pre-symptomatic rate	0.200	[2,3]
$\gamma_{1}$	Recovery rate	0.100	[2,3]
$\pi_{1}$	Proportion of severe cases	0.30	[2,3]
${ℛ}_{0}$	Basic Reproduction Number	**2.36**	Calculated

**Fig 5:**
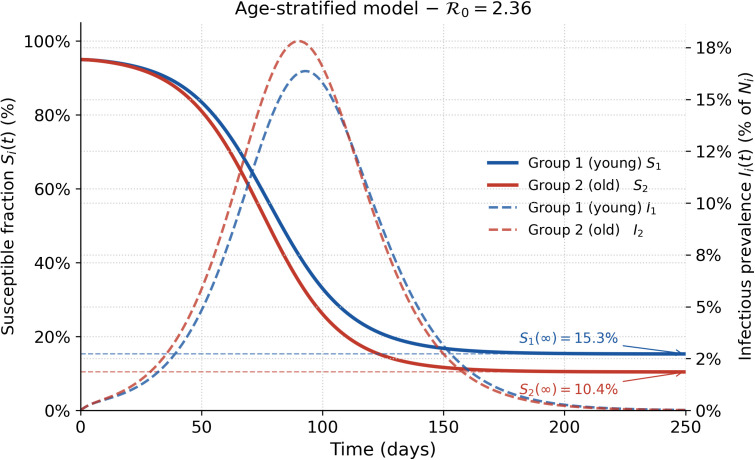
Scenario 4: Two-group age-stratified model (${ℛ}_{0}=2.36$). Solid lines: susceptible fractions $S_{i}(t)$ (left axis). Dashed lines: infectious prevalences $I_{i}(t)$ (right axis). Blue: young group (*p*_1_ = 80%); red: old group (*p*_2_ = 20%). Horizontal dashed lines mark the final susceptible fractions $S_{1}(\infty )\approx 15.3\%$ and $S_{2}(\infty )\approx 10.4\%$.

Fig 5 illustrates the key structural predictions of Theorem 5.1 and Remark 5.3. Several observations are noteworthy.

First, the two groups exhibit markedly different epidemic trajectories despite sharing the same ${ℛ}_{0}=2.36$: the young group (*p*_1_ = 80%) drives the epidemic through its higher contact rate *c*_11_ = 8, reaching a larger infectious peak earlier, while the old group experiences a delayed and smaller infectious peak due to lower intra-group contacts (*c*_22_ = 4) but substantial cross-group exposure.

Second, the final attack rates are group-specific: ${AR}_{1}\approx 83.9\%$ for the young group and ${AR}_{2}\approx 89.0\%$ for the old group. These high and similar attack rates reflect the assortative structure of the contact matrix (diagonal dominance) and confirms the theoretical result that $S_{i}(\infty )$ depends on the attack rates of *all* groups via the coupled transcendental system (56).

Third, the susceptible fractions converge monotonically to their respective limits $S_{1}(\infty )$ and $S_{2}(\infty )$ (horizontal dashed lines), consistent with the permanent immunity assumption and the global convergence result of Theorem 5.1. The effective reproduction number satisfies ${ℛ}_{0}(S(\infty ))<1$ at the end of the epidemic in both groups, confirming network-wide herd immunity.

These results underscore a key public health implication: protecting the high-risk old group cannot be achieved solely by targeting that group. Controlling transmission in the high-contact young group — which acts as the primary driver of the summation term in (56) — is essential for reducing the overall epidemic burden across the network [10,11].

## 7. Conclusion

This paper provides a rigorous mathematical analysis of a high-dimensional (13-compartment) Kermack–McKendrick-like model incorporating Erlang-distributed clinical delays and waning immunity. The model, adapted from the Institut Pasteur COVID-19 framework [3], is representative of a broad class of clinically detailed epidemic models for which the basic qualitative theory (stability, final size, endemic equilibrium) had not previously been established in full generality. The principal novelty of this work is threefold: (i) we derive explicit closed-form results for ${ℛ}_{0}$, final epidemic size, and endemic equilibrium for a 13-dimensional system, exploiting the DAG structure (H2) to show that hospitalization and ICU parameters do not affect the transmission-level quantities; (ii) we establish local stability of the endemic equilibrium and characterize the spiral-sink dynamics via an explicit discriminant condition (Theorem 4.11), a result that is new for Erlang-chained SEIRS systems; (iii) we extend all results to an age-stratified 13*n*-dimensional system via a Perron–Frobenius fixed-point argument. The main results are:

1. Permanent immunity. The Disease-Free Equilibrium is globally asymptotically stable if and only if ${ℛ}_{0}\leq 1$, and the model admits a unique final epidemic size $S_{\infty }$, characterised by a transcendental equation depending only on ${ℛ}_{0}$ and the initial conditions. This invariance with respect to the clinical delay parameters (Erlang stages, hospitalization rates) is a structural consequence of the DAG assumption H2: the clinical chain does not feed back into transmission.
2. Waning immunity. The DFE remains GAS when ${ℛ}_{0}\leq 1$ (Theorem 4.1). For ${ℛ}_{0}>1$, a unique endemic equilibrium exists and is locally asymptotically stable, with convergence occurring through damped oscillations (spiral sink, Theorem 4.11). Global stability of the endemic equilibrium remains an open problem.
3. Age stratification. The fundamental properties — uniqueness of the final size vector, network herd immunity threshold, and convergence to the DFE with ${ℛ}_{0}(S_{\infty })<1$ — extend to the multigroup setting under irreducibility of the contact matrix 𝒞 (Theorem 5.1). The heterogeneous model predicts markedly different group-specific attack rates compared to the homogeneous reference, with direct implications for age-targeted vaccination strategies [10,11].

From a methodological standpoint, the key contribution is the spectral decomposition of the 13×13 Jacobian at the endemic equilibrium via a cofactor expansion (Lemma 4.6), which reduces the stability question to a 6×6 epidemiological block $J_{ℱ}$. This technique may be useful for other high-dimensional compartmental models with similar cascade structures.

Relative to the recent literature on non-Markovian epidemic models [24–26], our contribution is complementary: while Basnarkov et al. [26] and Granger et al. [24,25] develop integro-differential frameworks that accommodate arbitrary sojourn distributions (including mortality, network heterogeneity, and stochastic effects), our Erlang-2 chain is the ODE-exact realization of the Gamma-(2,γ) kernel (Proposition 2.1), yielding explicit closed-form results for ${ℛ}_{0}$, final size, and equilibrium stability that remain analytically elusive in the general framework. The connection to network heterogeneity established by Pastor-Satorras and Vespignani [27] is captured in our age-stratified model through the spectral formula ${ℛ}_{0}=\alpha (q_{I},q_{s},q_{m})\;\rho (diag(S^{0})C)$, where the social contact matrix *C* plays the role of the network adjacency matrix.

**Limitations.** The principal limitations of the present analysis are the following.

(i) The model is formulated with three distinct transmission rates $\beta_{I},\beta_{s},\beta_{m}$ for the pre-symptomatic, severely symptomatic, and mildly symptomatic infectious compartments, respectively. The well-posedness, next-generation matrix, final-size relation, and disease-free stability results are established for the full three-rate formulation. The detailed local stability analysis of the endemic equilibrium is carried out under the calibration constraint $\beta_{I}=\beta_{s}=\beta_{m}=:\beta$, which is the parameterization used in the numerical simulations. However, the three rates are not *jointly identifiable* from aggregate hospitalization surveillance data alone [35]: only the compound quantity ${ℛ}_{0}=S_{0}(\beta_{I}/\gamma_{I}+(\beta_{s}\pi_{1}+\beta_{m}(1-\pi_{1}))/\gamma_{1})$ can be inferred without individual-level transmission data. In the numerical simulations we therefore impose the calibration constraint $\beta_{I}=\beta_{s}=\beta_{m}=:\beta$, consistent with the reference model [3]. Relaxing this constraint using contact-tracing or household-level data [34] is a natural next step, but lies outside the scope of the present mathematical analysis.
(ii) The global stability of the endemic equilibrium (Section 4) remains an open problem; local stability and the spiral-sink property are established, but a global Lyapunov argument for the full 13-dimensional system is not yet available.
(iii) Vital dynamics (births and natural deaths) are excluded by H1 (no vital dynamics); their inclusion would break the conserved quantity *N* and likely introduce backward bifurcation in the SEIRS case.
(iv) The model is deterministic; stochastic effects may be significant in the early growth phase or in small populations.
(v) The Erlang-2 sojourn time is a parsimonious approximation; Erlang-*k* with *k* > 2 or non-Erlang distributions (Weibull, log-normal) could improve empirical fit at the cost of higher model dimension or non-ODE formulations [24,25].
(vi) Age-dependent clinical severity parameters $\left(\pi_{1},\pi_{2}\right)$ and seasonal forcing are absent, limiting applicability to long-term endemic scenarios.

**Future directions** include: (a) the global stability of the endemic equilibrium via Lyapunov–Volterra methods adapted to Erlang-chained systems [40]; (b) incorporation of demographic turnover and seasonal forcing to model interannual epidemic cycles; (c) joint identifiability analysis of the three transmission rates $\left(\beta_{I},\beta_{s},\beta_{m}\right)$ using contact-tracing or household-level data, to test the calibration constraint $\beta_{I}=\beta_{s}=\beta_{m}$ empirically; (d) optimal vaccination and non-pharmaceutical intervention control strategies in the age-stratified framework using Pontryagin’s maximum principle; (e) systematic confrontation with COVID-19 hospitalization time-series data for parameter identification and model validation [42]; (f) extension to Erlang-*k* with *k* > 2 sojourn distributions using the integro-differential formulation of [24,25] and comparison with the ODE chain-trick version.

## Supporting information

S1 File Simulation code. This file contains the Python scripts used to perform the numerical simulations and generate the figures presented in the manuscript. The model parameters and initial conditions used for these simulations are provided in the tables within the main text.
(PY)

S1 Appendix Appendix. (PDF)
