# Supplementary material for: Dynamic properties of Kermack-McKendrick-like models
Source: PLoS One. 2026 Jul 14;21(7):e0352960. doi: 10.1371/journal.pone.0352960 (PMC13367913; doi:10.1371/journal.pone.0352960)
Supplement: S1 Appendix — (PDF) [file pone.0352960.s002.pdf]

A Proof of Theorem 3.3 (Global convergence to the DFE)

This appendix contains the detailed proof of Theorem 3.3.

Since $\dot{S}(t) = -(\beta_I I + \beta_s I_s + \beta_m I_m)S \le 0$, the function $S(t)$ is non-increasing. Being bounded below by 0, it converges to a limit $S_\infty \ge 0$. Integrating $\dot{S}$ over $[0, \infty)$:

$$S_0 - S_\infty = \int_0^\infty (\beta_I I(t) + \beta_s I_s(t) + \beta_m I_m(t))S(t)\,dt < \infty. \tag{69}$$

Since the total population is bounded and the vector field is smooth, the derivatives $\dot{I}, \dot{I}_s, \dot{I}_m$ are bounded, implying $I(t), I_s(t), I_m(t)$ are uniformly continuous on $[0, \infty)$. The function $g(t) = (\beta_I I(t) + \beta_s I_s(t) + \beta_m I_m(t))S(t)$ is therefore uniformly continuous. By Barbalat's Lemma [17], since $\int_0^\infty g(t)\,dt < \infty$ and g is uniformly continuous:

$$\lim_{t\to\infty} (\beta_I I(t) + \beta_s I_s(t) + \beta_m I_m(t))S(t) = 0.$$

Since $S_\infty > 0$ (the epidemic cannot infect the entire population in finite time), we conclude $\lim_{t\to\infty}(I + I_s + I_m) = 0$, hence $I(t), I_s(t), I_m(t) \to 0$.

For convergence of E: compute $\ddot{E} = (\dot{\beta_I} I + \dot{\beta_s} I_s + \dot{\beta_m} I_m)S + (\beta_I \dot{I} + \beta_s \dot{I}_s + \beta_m \dot{I}_m)S + (\beta_I I + \beta_s I_s + \beta_m I_m)\dot{S} - \gamma_E \dot{E}$. Since all rates are constant and $\dot{I} + \dot{I}_s + \dot{I}_m = \gamma_E E - \gamma_1(I_s + I_m)$, all terms are bounded, so $\ddot{E}$ is bounded and $\dot{E}$ is uniformly continuous. Since $\dot{S} + \dot{E} = -\gamma_E E$, the sum $S + E$ is non-increasing and bounded below, so $\dot{E}$ is integrable. By Barbalat's Lemma, $\dot{E} \to 0$; since $(\beta_I I + \beta_s I_s + \beta_m I_m)S \to 0$, we get $\gamma_E E \to 0$, hence $E \to 0$.

For the downstream compartments: the hospital sub-system vector $Y_H = (I_h, H_1, H_2)^T$ satisfies $\dot{Y}_H = M_H Y_H + U(t)$ where M_H is a stable triangular matrix and $U(t) \to 0$ as $I_s(t) \to 0$. By standard linear systems theory, $Y_H \to 0$. Similarly for the ICU chain. Finally, by conservation of mass, $R(t) = N - S(t) - \sum_{\text{infected}}(t) \to N - S_\infty$. □

B Proof of uniqueness of the final size S_∞

This appendix provides the proof of uniqueness of the solution to the final-size equation.

Define:

$$T(S_\infty) = S_\infty - S_0 - \frac{S_0}{R_0}(\ln S_\infty - \ln S_0) - \alpha_0,$$

where $\alpha_0 = E_0 + I_0 + \frac{(\beta_s I_{s,0} + \beta_m I_{m,0})\,S_0}{\gamma_1 \mathcal{R}_0\, S_0/\beta_I} \ge 0$, which under the calibration constraint $\beta_I = \beta_s = \beta_m =: \beta$ reduces to $\alpha_0 = E_0 + I_0 + \frac{\beta}{\gamma_1}\frac{S_0}{\mathcal{R}_0}(I_{s,0} + I_{m,0})$. We need a unique root in $(0, S_0/\mathcal{R}_0]$.

$T'(S_\infty) = 1 - S_0/(R_0 S_\infty)$, which vanishes at $S_\infty = S_0/R_0$. Thus T is strictly decreasing on $(0, S_0/R_0]$.

Boundary behavior: $\lim_{S_\infty\to 0^+} T(S_\infty) = +\infty$ (since $-\ln S_\infty \to +\infty$) and $T(S_0) = -\alpha_0 \le 0$.

Case 1 ($R_0 \le 1$): $(0, S_0/R_0] \supseteq (0, S_0]$. Since T is decreasing with $T(0^+) = +\infty$ and $T(S_0) \le 0$, the IVT gives a unique root in $(0, S_0] \subseteq (0, S_0/R_0]$.

Case 2 ($\mathcal{R}_0 > 1$): $S_0/\mathcal{R}_0 < S_0$. T is decreasing on $(0, S_0/\mathcal{R}_0]$ with $T(0^+) = +\infty$ and $T(S_0/\mathcal{R}_0) \le 0$. Unique root follows. Finally,

$$\mathcal{R}_0(S_\infty) = S_\infty\left(\frac{\beta_I}{\gamma_I} + \frac{\beta_s \pi_1 + \beta_m(1 - \pi_1)}{\gamma_1}\right) \le 1$$

since $S_\infty \le S_0/\mathcal{R}_0$. □

C Derivation of the clinical cascade transfer function $f(\lambda)$

This appendix provides the explicit computation of $f(\lambda) = \mathbf{e}^T(A - \lambda I_7)^{-1}\mathbf{c}_{I_s}$ appearing in equation (44), where $\mathbf{e}^T = (0, 0, 0, \gamma_H, 0, 0, \gamma_i)$,
$\mathbf{c}_{I_s} = (\pi_2\gamma_1,\, (1-\pi_2)\gamma_1,\, 0, \dots, 0)^T$, and A is the 7×7 Hurwitz matrix of the clinical sub-system (equation (41)).

Since A is lower-triangular with diagonal $(-\gamma_{I_h}, -\gamma_{I_i}, -\gamma_H, -\gamma_H, -\gamma_{H_i}, -\gamma_i, -\gamma_i)$, the resolvent $(A - \lambda I_7)^{-1}$ is also lower-triangular and its entries are computed by forward substitution. Using the two-pathway structure of the clinical chain (general ward: $I_h \to H_1 \to H_2 \to R$; ICU: $I_{\text{icu}} \to H_{\text{icu}} \to \text{ICU}_1 \to \text{ICU}_2 \to R$), one obtains:

Hospital pathway. A particle entering I_h passes through H_1 and then H_2 before contributing to $\dot{R}$ at rate γ_H. Tracing the chain:

$$[(A - \lambda I_7)^{-1}]_{I_h \to H_2} = \frac{\gamma_{I_h}\,\gamma_H}{(\lambda + \gamma_{I_h})(\lambda + \gamma_H)^2}.$$

The contribution of this pathway to $f(\lambda)$ is the product of the entry of $\mathbf{c}_{I_s}$ at I_h (namely $\pi_2\gamma_1$), the resolvent entry above, and the H_2 component of $\mathbf{e}$ (namely γ_H):

$$f_H(\lambda) = \pi_2\gamma_1 \cdot \frac{\gamma_{I_h}\,\gamma_H}{(\lambda + \gamma_{I_h})(\lambda + \gamma_H)^2} \cdot \gamma_H = \frac{\pi_2\,\gamma_1\,\gamma_{I_h}\,\gamma_H^2}{(\lambda + \gamma_{I_h})(\lambda + \gamma_H)^2}. \tag{70}$$

ICU pathway. A particle entering I_{icu} passes through H_{icu}, ICU_1, and ICU_2 before contributing to $\dot{R}$ at rate $\gamma_i = \gamma_{\text{icu}}$:

$$[(A - \lambda I_7)^{-1}]_{I_{\text{icu}} \to \text{ICU}_2} = \frac{\gamma_{I_i}\,\gamma_{H_i}\,\gamma_i}{(\lambda + \gamma_{I_i})(\lambda + \gamma_{H_i})(\lambda + \gamma_i)^2}.$$

The contribution to $f(\lambda)$ from the ICU pathway is:

$$f_U(\lambda) = (1-\pi_2)\gamma_1 \cdot \frac{\gamma_{I_i}\,\gamma_{H_i}\,\gamma_i}{(\lambda + \gamma_{I_i})(\lambda + \gamma_{H_i})(\lambda + \gamma_i)^2} \cdot \gamma_i = \frac{(1-\pi_2)\,\gamma_1\,\gamma_{I_i}\,\gamma_{H_i}\,\gamma_i^2}{(\lambda + \gamma_{I_i})(\lambda + \gamma_{H_i})(\lambda + \gamma_i)^2}. \tag{71}$$

Summing both pathways gives:

$$f(\lambda) = f_H(\lambda) + f_U(\lambda) = \frac{\pi_2\,\gamma_1\,\gamma_{I_h}\,\gamma_H^2}{(\lambda + \gamma_{I_h})(\lambda + \gamma_H)^2} + \frac{(1-\pi_2)\,\gamma_1\,\gamma_{I_i}\,\gamma_{H_i}\,\gamma_i^2}{(\lambda + \gamma_{I_i})(\lambda + \gamma_{H_i})(\lambda + \gamma_i)^2}, \tag{72}$$

which is equation (44). The function f is a sum of partial fractions with poles at $\{-\gamma_{I_h}, -\gamma_H, -\gamma_{I_i}, -\gamma_{H_i}, -\gamma_i\} \subset \sigma(A) \subset \{\text{Re} < 0\}$, is strictly positive at $\lambda = 0$, and satisfies $|f(\lambda)| \to 0$ as $|\lambda| \to \infty$ on $\{\text{Re}(\lambda) \geq 0\}$, as used in the proof of Theorem 4.8. $\square$
